# Oxylipins Associated to Current Diseases Detected for the First Time in the Oxidation of Corn Oil as a Model System of Oils Rich in Omega-6 Polyunsaturated Groups. A Global, Broad and in-Depth Study by ^1^H NMR Spectroscopy

**DOI:** 10.3390/antiox9060544

**Published:** 2020-06-20

**Authors:** Jon Alberdi-Cedeño, María L. Ibargoitia, María D. Guillén

**Affiliations:** Food Technology, Faculty of Pharmacy, Lascaray Research Center, University of the Basque Country (UPV-EHU), Paseo de la Universidad n° 7, 01006 Vitoria-Gasteiz, Spain; jon.alberdi@ehu.eus (J.A.-C.); marialuisa.ibargoitia@ehu.eus (M.L.I.)

**Keywords:** corn oil, linoleic acyl groups, oxidation, oxylipins, hydroproxy-, hydroxy-, keto-, epoxy-derivatives, aldehydes oxygenated *alpha,beta*-unsaturated, poly-formates, poly-hydroxy, poly-ethers, ^1^H NMR

## Abstract

For the first time, an important number of oxylipins have been identified and quantified in corn oil submitted to mild oxidative conditions at each time of their oxidation process. This oil can be considered as a model system of edible oils rich in polyunsaturated omega-6 groups. The study was carried out using ^1^H nuclear magnetic resonance spectroscopy (^1^H NMR), which does not require chemical modification of the sample. These newly detected oxylipins include dihydroperoxy-non-conjugated-dienes, hydroperoxy-epoxy-, hydroxy-epoxy- and keto-epoxy-monoenes as well as *E*-epoxy-monoenes, some of which have been associated with several diseases. Furthermore, the formation of other functional groups such as poly-formates, poly-hydroxy and poly-ether groups has also been proven. These are responsible for the polymerization and increased viscosity of the oil. Simultaneously, monitoring of the formation of well-known oxylipins, such as hydroperoxy-, hydroxy-, and keto-dienes, and of different kinds of oxygenated-*alpha,beta*-unsaturated aldehydes such as 4-hydroperoxy-, 4-hydroxy-, 4-oxo-2*E*-nonenal and 4,5-epoxy-2*E*-decenal, which are also related to different degenerative diseases, has been carried out. The provided data regarding the compounds identification and their sequence and kinetics of formation constitute valuable information for future studies in which lipid oxidation is involved, both in food and in other scientific fields.

## 1. Introduction

Lipid oxidation is one of the main degradative processes occurring in foods in general and in oil and fats in particular, with serious repercussions for food shelf-life, nutrition, and health. Oils in food processing are submitted to very varied conditions, in both industrial and culinary processes, during which their main and minor components might be degraded [1,2,3,4,5,6,7,8,9,10]. Likewise, some studies have also suggested that during gastrointestinal digestion, oxidation reactions take place, in which lipidic components undergo degradation [11,12,13,14]. Furthermore, it is well known that inside humans, many endogenous processes take place at the cellular level, in which lipid oxidation occurs [15,16,17].

Many factors influence lipid oxidation, among which the following can be cited: unsaturation degree of lipids, oxygen concentration in the system, temperature, light irradiation and the presence and concentration of minor compounds or molecular species able to exhibit either antioxidant or prooxidant activities [7,10,17,18]. In addition to the many influencing factors, the lipid oxidation reaction itself is a very complex process, which triggers a set of reactions, some simultaneous and others successive [19], giving rise to the formation of a large number of compounds of different nature, some of which remain still today unidentified. The great number of influencing factors and the numerous simultaneous and successive reactions involved have made it very difficult to know in-depth the predominant mechanisms through which lipid oxidation evolves in each system [19]. Due to the great importance of this degrading process, not only because it produces the deterioration of food but also because it generates toxic compounds [20,21,22,23,24,25], it has been the subject of many studies in both food and biological systems. The oxidation compounds formed in biological systems coming from polyunsaturated fatty groups are named generically as oxylipins. Although some of them play essential roles in normal physiology and function, others can provoke deleterious effects. Most of the well-known oxylipins derived from linoleic groups are also formed in the oxidation of edible oils [9,25,26]. However, in spite of this, many aspects of this highly complex reaction, such as the mechanisms through which it evolves, the identity and concentration of many of the compounds formed, and the evolution of some of their components, are not known nowadays.

Among lipidic foods, edible oils can be considered very appropriate models to study the above-mentioned subjects concerning lipid oxidation. They are of primordial importance for the food industry from technological, economical, nutritional, and safety points of view, and the study of their oxidation processes can help to enter in-depth into the oxidation that takes place in biological systems. Many of the studies regarding lipid oxidation in foods have been carried out by using classical methods that require chemical modification of the sample and provide very limited information about the identity of compounds whose functional groups are measured. Among these, both peroxide value (PV) as well as conjugated dienes (CD) to evaluate the occurrence of primary oxidation compounds, and thiobarbituric acid-reactive substances (TBARS) test to evaluate the occurrence of secondary oxidation products, are widely used. These methods only give information about certain compounds or functional groups and do not provide information about the specific nature of the compounds involved in each determination, which sometimes could give rise to inaccurate conclusions [27,28].

Furthermore, the study of the oxidation of edible oils has also been tackled by means of chromatographic techniques, such as gas chromatography (GC) and high-performance liquid chromatography (HPLC), coupled to different detectors. With these techniques, the study of volatile and non-volatile compounds, even of dimers, oligomers, and polymers formed during different thermo-oxidative processes [10,29,30,31,32,33,34] has been carried out.

In addition, the use of ^1^H NMR spectroscopy to these ends has been gaining prominence in the last twenty years. From the early studies in which its usefulness to characterize edible oils and to quantify their unsaturation degree and their molar percentage of the different kinds of acyl groups [35,36] was proved, notable advances have been made in the study of edible oil oxidation processes. These advances refer not only to the evolution of the edible oil oxidation process under different degradative conditions but also to the identification and quantification of new oxidation compounds, many of which are very relevant due to their negative bioactive properties [3,4,5,6,7,9,22,23,32,37,38,39] Despite these important advances, there are still many unknown aspects related to the complex process of oxidation of edible oils that can be discovered and understood using this technique.

In this context, the aim of this study is to analyze in a global, broad and in-depth way, by means of ^1^H NMR spectroscopy, the evolution of corn oil oxidation when it is submitted to mild oxidative conditions, similar to those of accelerated storage. Attention will be paid to the degradation rate not only of their main but also of their minor components. Moreover, simultaneously, the course of the oxidation will be addressed through the identification and formation rate of newly formed oxidation compounds as well as the degradation of some of these to give rise to secondary or further oxidation compounds. It is to be expected that new information could be extracted that will provide new insights into the oxidation of corn oil, which could be applied to the oxidation process of edible oils in general and also of biological systems. In addition, information provided in previous studies on oxidation of pure compounds, and also on certain processes applied to vegetable oils, will be taken into account. The interest of the methodology used lies in that it does not involve chemical modification of the sample and in just one run allows monitoring of a great number of compounds involved in the process, in a very short period of time, providing information about their concentration at any step of the process.

## 2. Materials and Methods

### 2.1. Samples Subject of Study

#### 2.1.1. Original Oil

The study was carried out with refined corn oil, purchased in a local supermarket (Vitoria-Gasteiz, Spain). Its composition in acyl groups was determined from ^1^H NMR spectral data as in previous studies [36,38,40]. The molar percentages of the different kinds of acyl groups, regarding the total of acyl groups, were linolenic group, Ln, 0.6 ± 0.0%; linoleic group, L, 48.7 ± 0.0%; oleic group, O, 33.0 ± 0.1%; and saturated group, S, 17.7 ± 0.1%. These compositional data can be also expressed in mmol regarding the number of moles of triglyceride, TG; in this way, this oil contains 18.8 ± 0.0 mmoles of Ln/mol TG, 1461.6 ± 0.1 mmoles of L/mol TG, 990.2 ± 1.7 mmoles of O/mol TG, and 529.4 ± 1.5 mmoles of S/mol TG.

#### 2.1.2. Oxidized Oil Samples

In addition to the original oil, samples derived from this oil, after their submission to mild oxidative conditions similar to those of accelerated storage for different periods of time, were also the subject of study. To prepare these derived samples, 10 g amounts of original corn oil were placed in glass Petri dishes (80 mm in diameter and 15 mm deep) and kept in an oven at 70 °C with aeration over different periods of time of up to sixteen days. Under these conditions, the progress of the oxidative process in each sample increased in line with the time during which each sample was kept under oxidative conditions, thus allowing a continuous view of the process from its beginning to the total polymerization of the sample. These experiments were performed in duplicate to obtain sound results. 

### 2.2. Acquisition of the ^1^H Nuclear Magnetic Resonance Spectra (^1^H NMR)

#### 2.2.1. Operating Conditions

The ^1^H NMR spectra of the original oil and of the samples derived from this oil, after being subjected to degradative conditions, were acquired in duplicate using a Bruker Avance 400 spectrometer operating at 400 MHz. For this purpose, the above-mentioned samples (approximately 0.16 g) were dissolved in 400 µL of deuterated chloroform, which contained tetramethylsilane (TMS) as internal reference (Cortec, Paris, France). The acquisition conditions were the same as those used in previous studies [1,41]. The relaxation delays and acquisition times allow the complete relaxation of the protons, the areas of the signals thus being proportional to the number of protons that generate them, making their use for quantitative purposes possible. The ^1^H NMR spectra were plotted at a fixed value of absolute intensity to be valid for comparative purposes using the MestreNova program (Mestrelab Research, Santiago de Compostela, Spain).

#### 2.2.2. Identification of Components

Identification, both of components present in the original oil and of components generated in the oxidation process, was carried out on the basis of the assignments of the ^1^H NMR signals to the different kinds of hydrogen atoms and to the different compounds. These signals, which are shown in different figures, their chemical shifts and their assignments to the several hydrogen atoms are given in Tables S1–S8 (Supplementary Material). The assignments were made taking into account previous studies as indicated in Tables S1–S8 (Supplementary Material), or on the basis of the signals of standard compounds acquired for this study. Among these latter are: Δ5-avenasterol, sitostanol, acquired from ChemFaces Biochemical Co., LTD (Wuhan, China), β-sitosterol, (*E*)-2-hexenal, (*E*)-2-heptenal, (*E*)-2-decenal, (*E,E*)-2,4-hexadienal, (*E,E*)-2,4-heptadienal, (*E,E*)-2,4decadienal, 4,5-epoxy-(*E*)-2-decenal, 12,13-epoxy-9(*Z*)-octadecenoic acid methyl ester (isoleukotoxin methyl ester), 2-pentylfuran, 2-ethylfuran, amylformate and octylformate acquired from Sigma–Aldrich (St. Louis, MO, USA); 9,10-Epoxy-12-*Z*-octadecenoic acid (leukotxin), 4-hydroxy-(*E*)-2-nonenal, 4-hydroperoxy-(*E*)-2-nonenal, 4-oxo-(*E*)-2-nonenal, 9,10-dihydroxy-12-(*Z*)-octadecenoic acid (leukotoxin diol), 12,13-dihydroxy-9-(*Z*)-octadecenoic acid (isoleukotoxin diol), *trans*-12,13-epoxy-9-keto-10(*E*)-octadecenoic acid, 9-keto-10(*E*),12(*E*)-octadecadienoic acid, linolein hydroperoxides, linolein hydroxides, 9-oxo-10*E*,12*Z*-octadecadienoic acid, 13-oxo-9*Z*,11*E*-octadecadienoic acid and 12R-hydroxy-9(*Z*)-octadecenoic acid methyl ester (ricinoleic acid methyl ester), purchased from Cayman Chemical (Ann Arbor, MI, USA), Δ5-campesterol, Δ7-avenasterol, 9(S)-Hydroxy-10(*E*),12(*E*)-octadecadienoic acid (Dimorphecolic acid), Methyl 9(S),10(R)-epoxy-13(S)-hydroxy-11-(*E*)-octadecenoate, 9(S),10(S)-epoxy-11(S)-hydroxy12-(*Z*)-octadecenoic acid methyl ester, 11(S),12(S)-epoxy-13(S)-hydroxy-9(*Z*)-octadecenoic acid methyl ester, 9-hydroxy-10-oxo-12(*Z*)-octadecenoic acid; 9,10-12,13-diepoxyoctadecanoic acid and 13-hydroxy-12-oxo-9(*Z*)-octadecenoic acid acquired from Larodan (Malmö, Sweeden).

#### 2.2.3. Quantification of the Components

This was possible because the area of each ^1^H NMR spectral signal is proportional to the number of protons that generates it and because the proportionality constant is the same for all kinds of protons. Taking this into account, the estimation of the concentrations of the different functional groups, or of groups of compounds, as well as of minor components, regarding the concentration of triglycerides TG can be carried out by using the area of the corresponding spectral signals. Triglycerides can be considered as an internal reference due the low level of hydrolysis that these undergo during oxidation. To this aim, the Equation (1) was used.

(1) $$\left[X\right]=\frac{A_{x}/n}{A_{\mathrm{TG}}/4}\times 1000$$

In this equation, A_X_ is the area of the signal selected for the quantification of the X functional group, *n* is the number of protons that generate this signal and A_TG_ the area of the protons at *sn*-1 and *sn*-3 positions in the triglyceride backbone TG (signal TG in Table S1). In this way, the concentration obtained is expressed in millimole per mol of triglyceride (mmol/mol TG). The area of the signals used was determined by using the equipment software and the integrations were made three times to obtain average values.

### 2.3. Statistical Analysis

Data represented in the different figures and those given in Table S9 (Supplementary Material) are mean values obtained as average values of at least two determinations. Microsoft Office Excel 2007 was used for the statistical analysis and for the graphical representation of the obtained values. 

## 3. Results and Discussion

As mentioned before, this methodology allows one to monitor, throughout the oxidation process, the degradation rate of the original main oil components and also of minor ones. Furthermore, and simultaneously, this methodology permits the detection of new compounds formed as a consequence of the degradation of the former. In this study, the identification of the newly formed compounds will be addressed as will the estimation of their concentration and evolution during the oxidation process, with the aim of obtaining as complete and integrated as possible a picture of the whole oxidation process of this oil. 

### 3.1. Evolution of Original Oil Components

#### 3.1.1. Corn Oil Main Components

**Figure 1:**
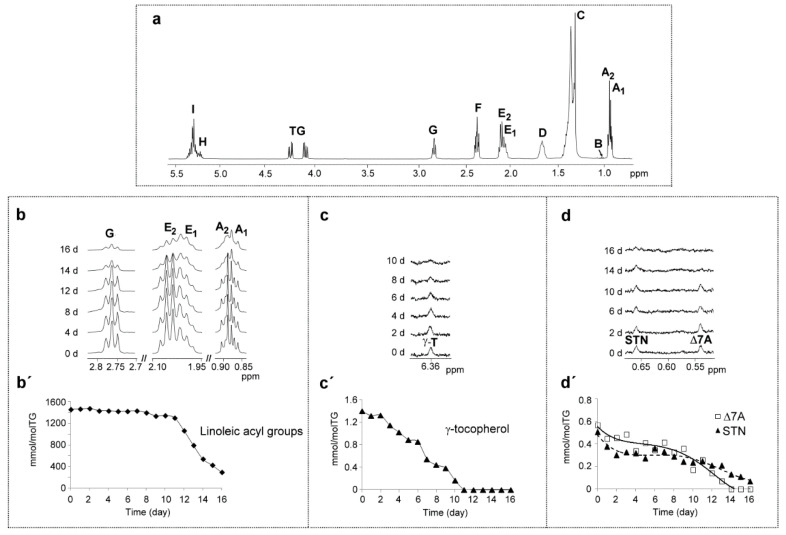
(**a**) Region between 0.5 and 5.5 ppm of corn oil, C, ^1^H NMR spectrum. Spectral regions, conveniently enlarged, of the signals of (**b**) methylic, *mono*-allylic and *bis*-allylic; (**c**) *gamma*-tocopherol (γ-T); (**d**) Δ7-avenasterol (Δ7A) and sitostanol (STN). The signal letters agree with those of Table S1 of Supplementary Material. Evolution of the concentration, expressed as mmol/mol TG, present in corn oil submitted to oxidation process versus time given in days of (**b′**) linoleic acyl groups, (**c′**) *gamma*-tocopherol (γ-T), (**d′**) Δ7-avenasterol (Δ7A) and sitostanol (STN).

As is well known, the main edible oil components are triglycerides-supporting acyl groups having different unsaturation degrees and chain lengths. In corn oil, the main acyl group is linoleic, as already indicated. For this reason and due to its unsaturation degree, this acyl group can be considered an appropriate representative for the study of the degradation rate of corn oil’s main components. The hydrogen atoms of this acyl group, as Table S1 and Figure 1a,b show, give several ^1^H NMR spectral signals, some of them specific to its methylic, *mono*-allylic and *bis*-allylic protons (in Figure 1b, signals A2, E2 and G respectively). As may be expected, the intensity of these signals decreases as the oxidation process advances (Figure 1b), and from the area of signal G, the evolution of the concentration of linoleic acyl group (or linoleate group) at different times under degradative conditions can be estimated by using Equation (1). This evolution has been represented versus time in Figure 1b′. It can be observed that after 16 days under degradative conditions, the corn oil has reached a very high polymerization degree, and the linoleic group is in a very low concentration (near 291.2 ± 29.5 mmol/mol TG).

#### 3.1.2. Corn Oil Minor Components

As is well known, corn oil has different kinds of minor components [42,43]. Among them, tocopherols and sterols-stanols are important because they have been attributed antioxidant activity and also because their concentrations are higher than those of other minor corn oil components.

##### (a) Regarding Tocopherols

It is known that corn oil contains *α-, β-, γ*- and *δ*-tocopherol [42]. Two conditions are required for their study to be carried out by ^1^H NMR: that their spectra have at least one signal that does not overlap with any other, and that their concentrations in the oil are high enough to be able to be detected by ^1^H NMR spectroscopy. Among the tocopherols present in this corn oil, only *γ*-tocopherol meets these two required conditions. The ^1^H NMR spectrum of this compound has a signal of its aromatic proton [44] (signal γT at 6.360 ppm in Figure 1c and in Table S1) that does not overlap with any other. It can be observed in Figure 1c that the intensity of this signal decreases during the oxidation process until its total disappearance. The concentration of this compound, determined by Equation (1), is represented versus time in Figure 1c′. It can be observed that the degradation of *γ*-tocopherol throughout this oxidative process has two stages, which is in agreement with observations from in a previous study carried out by direct immersion-solid-phase microextraction followed by gas chromatography / mass spectrometry (DI–SPME–GC/MS) [10]. In the first stage (days 0-2), the concentration of this compound, initially near 1.4 ± 0.0 mmol/mol TG, remains almost unchanged, its rate of degradation was near to 0.04 mmol/mol TG per day. However, in a second stage, from day 2 to 11, a significant decrease took place, with an estimated degradation rate near 0.14 mmol/mol TG per day. On day 11, its presence could not be detected by ^1^H NMR, the reason for which it could be said that it had practically disappeared from the oil. These results suggest that in the period of time in which there is *γ*-tocopherol in the oil sample, the degradation rate of linoleic group is low (near 14 mmol/mol TG per day), and when this compound disappears, this rate increases very sharply. However, it must be pointed out that the presence of *γ*-tocopherol in the oil does not prevent the degradation of either the linoleic group or indeed of the corn oil, although its presence slows down its degradation.

##### (b) Regarding Sterols-Stanols

It is well known that corn oil can contain brassicasterol, ∆5-stigmasterol, β-sitosterol, ∆5-campesterol, sitostanol and ∆7-avenasterol [42]. Among all these compounds, only sitostanol (STN) and ∆7-avenasterol (Δ7A) have, at least, a ^1^H NMR signal that does not overlap with any other (Figure 1d and Table S1) and are in enough concentration to be detected by ^1^H NMR. This signal, which is centered at 0.651 ppm in the case of STN [45] and at 0.540 ppm in the case of Δ7A [45,46,47] (Figure 1d and Table S1), is due in both cases to their methylic protons on the C18. It can be observed in Figure 1d that the intensity of these signals remains almost unchanged up to the last days of the experiment. The concentration of these compounds at different times under the degradative conditions was determined also by using Equation (1) and is depicted in Figure 1d′. The results indicate that these compounds, which are in much lower concentration than *γ*-tocopherol, have higher oxidative stability than the latter, and as a consequence they show a very small degradation rate. This increases somewhat from day 9 onwards, although both compounds are present in the sample up to days 13 (Δ7A) and 16 (STN. The higher oxidative stability of STN regarding Δ7A could be attributed to its lower unsaturation degree. In summary, at the end of this experiment, the only totally undegraded minor corn oil component of the two studied here is STN, even though its concentration has been significantly reduced. These results are in general agreement with those observed in this kind of compounds in a previous study using a very different technique [10].

### 3.2. Formation of New Compounds

As is well known, alongside the degradation of original oil main and minor components, new derived compounds are formed. In this study, attention is first paid to the formation of compounds derived from main components, and secondly to some compounds derived from oil minor components. Tables S2–S8 give the ^1^H NMR signals found in the spectra of corn oil at different times under degradative conditions, their chemical shifts and multiplicities, and their assignment to protons of different functional groups, structures supporting several functional groups or compounds that will be commented on below. Likewise, the quantification of the different functional groups or of structures supporting several functional groups will be made in all cases by using Equation (1).

#### 3.2.1. Compounds Derived from Corn Oil Main Components

The degradation of oil main components gives rise to the formation of so-called primary oxidation compounds, and the evolution of these latter gives rise to the formation of so-called secondary oxidation compounds and so on. ^1^H NMR spectroscopy allows one to detect them, to estimate their concentration, and to follow their evolution through the oxidation process.

##### (a) Monohydroperoxides (mHPOs)

**Figure 2:**
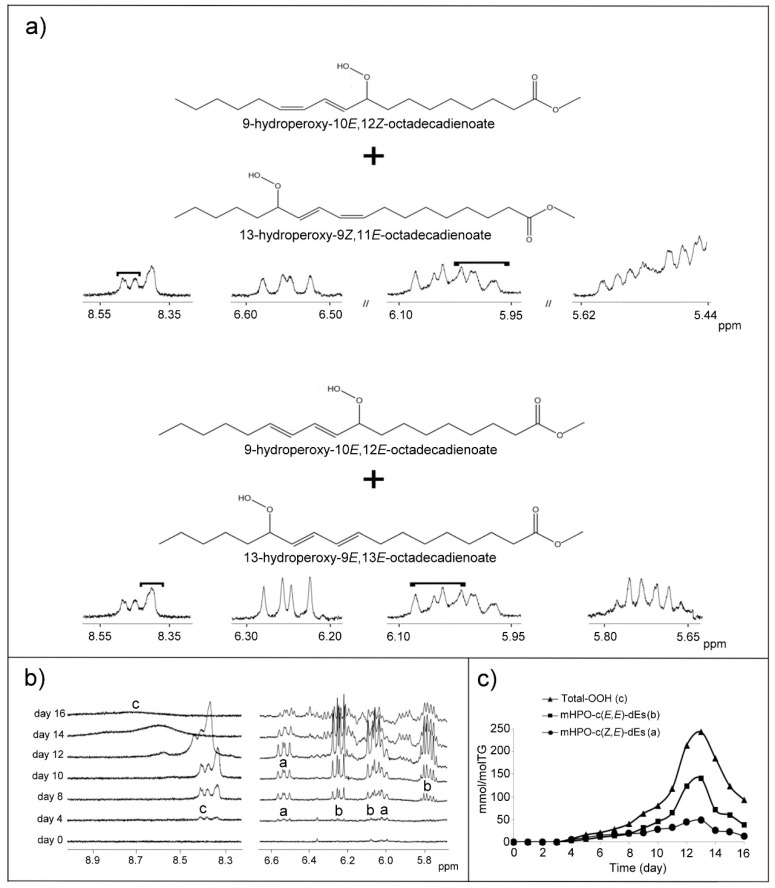
(**a**) Chemical structure of the monohydroperoxy-conjugated dienes (mHPO-c-dEs) detected in corn oil submitted to oxidative conditions, together with the enlargement of some regions of the ^1^H NMR spectra in which the signals of monohydroperoxy-conjugated dienes appear. (**b**) Enlargements of some spectral regions between 5.8 and 6.6 ppm and 8.3 and 8.9 ppm, where changes occur throughout the oxidation process and their evolution with time. (**c**) Evolution of the concentration of mHPO-c(*Z,E*)-dEs, mHPO-c(*E,E*)-dEs and Total-OOH, expressed as mmol/mol TG versus time given in days.

These are the first compounds detected by ^1^H NMR spectroscopy as being new compounds formed by the degradation of corn oil main components under these conditions. As mentioned above, the concentration of linolenic groups in this oil is very small, almost negligible, linoleic being the main unsaturated acyl group in this oil. For this reason, and taking into account the low oxidative stability of the linoleic group, the first main hydroperoxides detected should come from this latter acyl group. Indeed the first structures detected as new in this corn oil submitted to oxidative conditions, are monohydroperoxy-conjugated dienes, mHPO-c-dEs, derived from linoleic group; the well-known 9- and 13-hydroperoxy-*Z,E*-conjugated dienes (mHPO-c(*Z,E*)-dEs) and 9- and 13-hydroperoxy-*E,E*-conjugated dienes (mHPO-c(*E,E*)-dEs) (Figure 2 and Table S2). They can be easily identified and quantified from ^1^H NMR spectral data. Their identification is based on the signals of their conjugated olefinic protons and of the corresponding OOH group proton, which are shown in Figure 2a. Figure 2b shows the evolution of these spectral signals in the corn oil at different times. Their concentration can be determined separately over time using the area of their signals centered at 6.55 ppm in the case of *Z,E*-isomers and at 6.24 ppm in the case of *E,E*-isomers. Figure 2c shows the evolution of their concentrations over time. It can be observed that from day 4 to day 8, the concentration of the *Z,E*-isomers is somewhat higher than that of the *E, E*-isomers and the opposite happens from day 8 onwards. Both kinds of isomers reached the maximum concentration near day 13 (*E,E*-isomers near 140.9 ± 3.4 mmol/mol TG and *Z,E*-isomers near 48.5 ± 2.4 mmol/mol TG). After this maximum, the concentration of these compounds decreased very sharply until day 16, *E,E*- and *Z,E*-isomers reaching 37.9 ± 3.7 and 12.3 ± 3.0 mmol/mol TG, respectively. This diminution evidences their role as intermediate compounds.

Furthermore, from the beginning of the process up to day 8, the total concentration of hydroperoxy groups (Total-OOH), determined from the area of the signal of the OOH protons (between 8.38 and 8.52 ppm), was coincident with the sum of the mHPO-c-dEs above mentioned. However, from day 9 onwards, new signals of hydroperoxy protons appear clearly in the spectrum and as consequence, the concentration of total hydroperoxy groups is higher than the sum of the above-cited mHPO-c-dEs (Figure 2c). The new signals appear at higher and also at lower ppm values than those of the OOH protons of the aforementioned *Z,E*- and *E,E*-isomers. All these signals, in advanced oxidation stages, become broader and appear from 8.3 to 9.3 ppm, and they can be attributed, in addition to the above-mentioned conjugated isomers, to other compounds also supporting hydroperoxy groups. 

In this context, it could be thought that monohydroperoxy-non conjugated dienes, mHPO-nc-dEs could also be formed in this process. In fact, the formation of mHPO-nc-dEs supporting the hydroperoxy group in carbon atoms numbers 8 or 14 maintaining the double bonds (9*Z*,12*Z*) of linoleic group (8-hydroperoxy-9*Z*,12*Z*-octadecadienoate and 14-hydroperoxy-9*Z*,12*Z*-octadecadienoate) has been described in autoxidation processes of linoleate, although in very low concentrations (Figure S1a) [48]. It should be noted that although ^1^H NMR spectral data of these mHPO-nc-dEs have not been given, they maintain the *bis*-allylic protons of the original linoleic group and the chemical shifts of their spectral signals should be very similar to those of the original linoleic group. The proportion of these compounds in autoxidation processes has been estimated as about 1% of the total of mHPO formed [49]. For this reason, their detection, if they are formed under the conditions of this study, is very difficult if not impossible by the technique here used.

In addition, other mHPO-nc-dEs such as 10-hydroperoxy-8*E*,12*Z*-octadecadienoate and 12-hydroperoxy-9*E*,13*Z*-octadecadienoate (Figure S1a) have been reported as forming from linoleate groups only under photoinduced oxidative conditions [50], for which reason they could not be expected to form in the process here studied. This is in agreement with the absence in the spectra of proton signals of the hydroperoxy groups near 7.8–7.9 ppm described for these kinds of compounds [50] and is the reason for which their formation can be discarded in the process here studied.

Finally, the mHPO-nc-dE, 11-hydroperoxy-9Z,12Z-octadecadienoate (Figure S1a), has been described in the oxidation of linoleate groups, together with 9- and 13-mHPO-c-dEs, only when the oxidation occurs in presence of *alpha*-tocopherol [51], for which reason their occurrence should not be expected in this study.

##### (b) Dihydroperoxides (dHPOs)

In addition to mHPO-dEs, dihydroperoxydienes, dHPO-dEs, either conjugated (dHPO-c-dEs) and/or non conjugated (dHPO-nc-dEs) could also be formed by subsequent oxidation of mHPO-dEs. In fact, both kinds of dHPOs have been described as intermediate compounds in several formation pathways of aldehydes [52,53]. 

**Figure 3:**
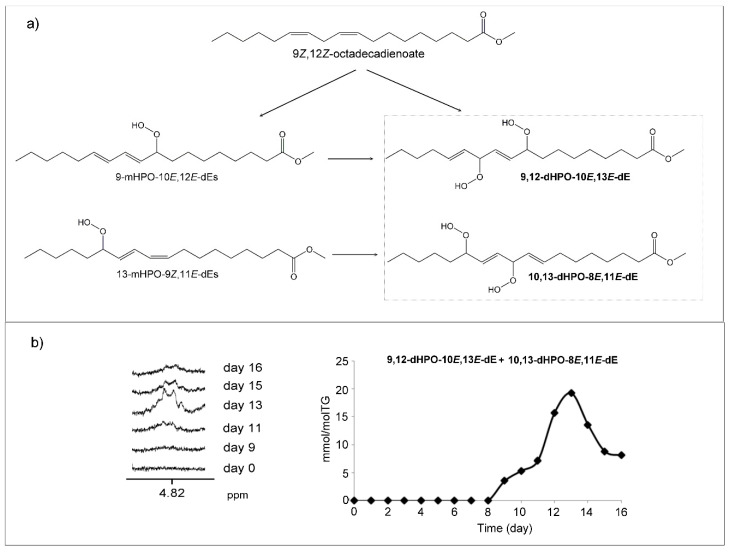
(**a**) Pathways of formation of dihydroperoxy-non conjugated dienes (dHPO-nc-dEs) proposed by some authors under certain oxidation conditions [50,54]. (**b**) Enlargements of the spectral region where changes occur throughout the accelerated storage process and their evolution with time, together with the graphical representation of the evolution of the concentration of 9,12-dHPO-10*E*,13*E*-dE + 10,13-dHPO-8*E*,11*E*-dE, expressed as mmol/mol TG, versus time given in days.

Regarding dihydroperoxy-non conjugated dienes dHPO-nc-dEs, some such as 9,12-dihydroperoxy-10*E*,13*E*-octadecadienoate (9,12-dHPO-10*E*,13*E*-dE) and 10,13-dihydroperoxy-8*E*,11*E*-octadecadienoate (10,13-dHPO-8*E*,11*E*-dE) have been described [54] (Figure 3a). Among their ^1^H NMR signals given in the literature [54], there is a double doublet near 4.82 ppm (Figure 3b) of one of the methine carbinol protons, being the signals of the rest of protons overlapping with those of other oxidation compounds. This signal appears in the spectra of the oil studied here from day 9 onwards and reaches its maximum intensity on day 13 like the mHPO-c-dEs, after which their intensity decreases, thus indicating their role as intermediate compounds. The estimated concentration of these potential dHPO-nc-dEs on day thirteen is near 19.2 ± 0.2 mmol/mol TG (Figure 3b). These compounds have been proposed as intermediate for the formation of different compounds, among which are certain dioxo derivatives [54]. 

In addition to the above-mentioned dHPO-nc-dEs, others could be formed by oxidation of mHPO-c-dEs coming from linoleate. It has been described that one of them, 10,12-dihydroperoxy-8*E*,13*E*-octadecadienoate (10,12-dHPO-8*E*,13*E*-dE) (Figure S1b), gives signals in the ^1^H NMR spectrum [50] near 8.05 and 8.13 ppm attributable to protons of the OOH groups. In the spectra of the oil studied here, from day 12 onwards, signals appear overlapping with other ones included in a very broad signal between 8.00 and 8.17 ppm, for which reason it could be thought that these hydroperoxides could be present in this corn oil. However, these signals are not due to hydroperoxy groups because they do not disappear in the spectra when deuterated water is added, making it evident that these hydrogen atoms are not exchanged for deuterium as occurs in hydroperoxy groups. Furthermore, other signals of this structure such as a multiplet at 5.83 ppm and a double doublet near 4.45 ppm are not clearly observed in the spectra. For all these reasons, the formation of this dHPO-nc-dE in this oxidation process cannot be guaranteed. 

Likewise, the formation of dihydroperoxy-conjugated dienes, dHPO-c-dEs, could be possible [55]. 8,13-dihydroperoxy-10*E*,12*E*-octadecadienoate (8,13-dHPO-10*E*,12*E*-dE) and 9,14-dihydroperoxy-11*E*,13*E*-octadecadienoate (9,14-dHPO-11*E*,13*E*-dE) (Figure S1b) coming from mHPO-c-dEs of linoleic groups has been described as possible under induced oxidation conditions. Some of these compounds have also been proposed as intermediates in the formation of toxic aldehydes, some of which are formed in the oxidation of corn oil, which will be discussed later. However, the ^1^H NMR signals of specific protons of these compounds provided by some authors [55] are not clearly distinguishable (7.82–8.25 (br, s), 4.87 (m), 4.57 (m), 4.40 (m), 4.36 (m), 4.30 (m) ppms) in the spectra of the samples studied here. This indicates that if they are formed, the concentration is not enough to be detected by the technique used here. 

Closely related with the above compounds are those having both hydroperoxy and peroxy cyclic groups of six members, also named hydroperoxy-epidioxy-monoene compounds (mHPO-EPIdO-mE). These could be formed in this oxidation process from linoleic group. Some of them, such as 13-hydroperoxy-9,12-epidioxy-10-octadecenoate (13-HPO-9,12-EPIdO-10-octadecenoate) and 9-hydroperoxy-10,13-epidioxy-11-octadecenoate (9-HPO-10,13-EPIdO-11-octadecenoate) (Figure S1c), whose ^1^H NMR signals have been described [55], either are absent in the sample’s subject of study or are in very small concentrations. This is deduced because although signals near 4.61 and 4.66 ppm could be present overlapped with other ones in the spectra, the other signals due to the proton bonded to the carbon atom supporting the hydroperoxy group centered at 4.14-4.12 ppm, and 4.17 ppm, respectively, are not clearly visible in the spectra of this oil submitted to mild degradative conditions.

##### (c) Hydroperoxy-Epoxy-Monoenes (HPO-EPO-mEs)

**Figure 4:**
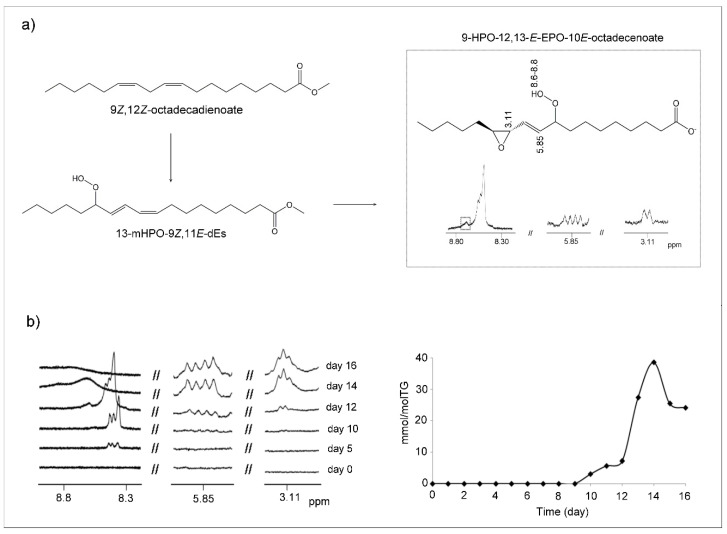
(**a**) Pathway of formation of hydroperoxy-epoxy-monoenes (HPO-EPO-mEs) proposed by some authors under certain oxidation conditions [56], together with some chemical shifts (ppm) of the ^1^H NMR signals of some of their hydrogen atoms. (**b**) Enlargements of some spectral regions where changes occur throughout the accelerated storage process and their evolution with time, together with the graphical representation of the evolution of the concentration of 9-HPO-12,13-*E*-EPO-10*E*-octadecenoate, expressed as mmol/mol TG, present in corn oil submitted to accelerate storage conditions, versus time given in days.

Other compounds which may be formed as a consequence of the oxidation of mHPO-c-dE are hydroperoxy-epoxy-monoenes (HPO-EPO-mEs) (Figure 4a). Studies about the formation of this kind of compounds in the oxidation of linoleic group [56] and of the analysis of their ^1^H NMR spectral signals are very scarce. In fact, to the best of our knowledge, only the ^1^H NMR signals of 9-HPO-12,13-*E*-EPO-10*E*-octadecenoate have been reported previously [56]. As Figure 4a and Table S2 show, signals of epoxydic protons (3.11 and 2.84 ppm), of olefinic proton in position *alpha* to the C-OOH group (double doublet at 5.85 ppm), and of hydroperoxy protons near 8.6–8.8 ppm are typical of the HPO-EPO-mE mentioned above [56]. All these signals, as Figure 4a shows, are present in the spectra of the corn oil here studied. They appear simultaneously from day 10 onwards (Figure 4b). It has been described that this compound is derived from the above-mentioned mHPO-c-dE, 13-mHPO-9Z,11*E*-octadecadienoate, and the possible formation mechanisms have also been explained [56]. 

Furthermore, the simultaneous appearance of a signal of epoxydic proton near 3.13 ppm is probably due to another isomer of the above mentioned HPO-EPO-mE, because both signals have the same evolution over time. These signals are visible in the ^1^H NMR spectra from day 10 onwards and appear almost simultaneously to that of dHPO-nc-dEs at 4.82 ppm, both being the first kinds of secondary oxidation compounds formed.

The confirmation of the assignment of the above signals to the afore-cited structure (Figure 4a) is corroborated by a more recent study in which several isomers having this structure have been found in the autoxidation of mHPO-c-dEs derived from the arachidonic group [57]. Nevertheless, the mechanism proposed in this latter study is somewhat different from that of other authors [56] because, although both cases involve an intermediate dimer, in the latter case the hydroperoxy group remains in the same position in HPO-EPO-mEs as in its corresponding precursor mHPO-c-dEs. In addition, these authors say that the HPO-EPO-mEs formed constitute near 20–30% of the total polar compounds derived from the oxidation of mHPO-c-dEs of the arachidonic group. This shows the relevance of these compounds as secondary oxidation compounds. 

The concentration of all these HPO-EPO-mEs over time was determined from the area of the double doublet centered near 5.85 ppm. The results indicate that these compounds that appear from day 10 onwards reach their highest concentration (near 38.7 ± 0.6 mmol/mol TG) near day 14, with a very small decrease on day 16 up to 24.2 ± 1.2 mmol/mol TG (Figure 4b). 

It only remains to add that, to the best of our knowledge, these oxidation compounds, which are considered inhibitors–uncouplers of mitochondrial respiration [58] have not been described before as forming in the oxidation of vegetable oils, except briefly in previous studies of our group [39].

##### (d) Monohydroxy-Conjugated Dienes (mHO-c-dEs)

**Figure 5:**
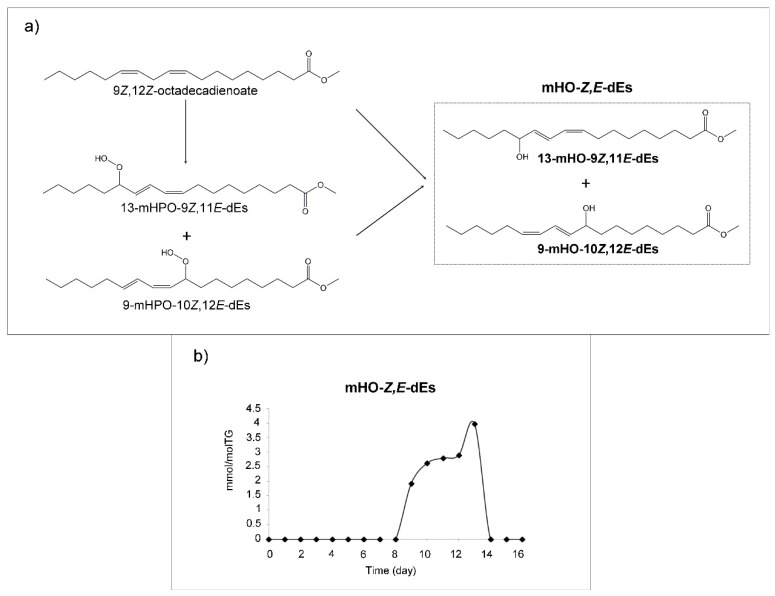
(**a**) Pathway of monohydroxy-conjugated-*Z,E*-dienes (mHO-c(*Z,E*)-dEs) formation proposed by some authors under certain oxidation conditions [61]. (**b**) Evolution of its concentration, expressed as mmol/mol TG versus time given in days.

From day 8 to day 13, signals of monohydroxy-conjugated-*Z,E*-dienes, centered at 6.48 ppms, with very low intensity, are observed. They can come from direct oxidation of linoleic groups or from reduction of mHPO-c-dEs or from peroxyl radicals by loss of an oxygen atom (Figure 5a). These are clearly intermediate compounds, and their signals are not visible from day 13 onwards. The evolution of their concentration is represented in Figure 5b. It must be noted that their low concentration requires a very detailed observation of the spectra in order to recognize the presence of these compounds. *E,E*-isomers may also form in very low concentrations as their precursors are present; however, their signals are not clearly visible in the spectra due to overlapping of their signals with others, for which reason they were not quantified. It should be added that these compounds have been shown to be cytotoxic [59], and together with other oxylipins have been associated with rheumatoid arthritis [60].

##### (e) Hydroxy-Epoxy-Monoenes (HO-EPO-mEs)

**Figure 6:**
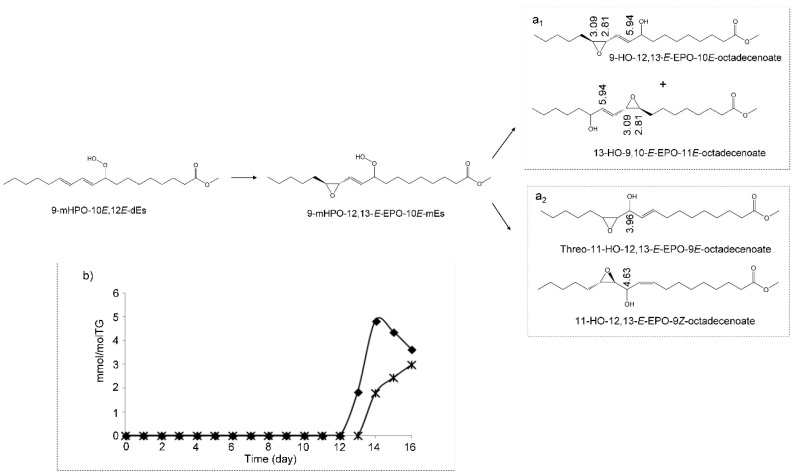
(**a**) Pathways of formation of hydroxy-epoxy-monoenes (HO-EPO-mEs) proposed by some authors under certain oxidation conditions [62,65] for (**a_1_**) mHO-EPO-mEs having the double bond between the epoxy and hydroxy groups together with some chemical shifts (ppm) of the ^1^H NMR signals of some of their hydrogen atoms; (**a_2_**) mHO-EPO-mEs having hydroxy and epoxy group vicinal together with some chemical shifts (ppm) of the ^1^H NMR signals of some of their hydrogen atoms. (**b**) Evolution of the concentration, expressed as mmol/mol TG versus time given in days of: 9-HO-12,13-*E*-EPO-10*E*-octadecenoate + 13-HO-9,10-*E*-EPO-11*E*-octadecenoate + 13-HO-9,10-*Z*-EPO-11*E*-octadecenoate (♦); *threo*-11-HO-12,13-*E*-EPO-9*E*-octadecenoate + *threo*-11-HO-9,10-*E*-EPO-12*E*-octadecenoate (*).

The formation of several kinds of HO-EPO-mEs has been described in the oxidation of linoleic acid or linoleate structures from HPO-EPO-mEs [56,62] as Figure 6a shows. In the oxidation process of this corn oil, signals assignable to two different groups of these compounds appear after 13 days under oxidative conditions. Nevertheless, some of their spectral signals overlap to a certain extent. 

The first group includes those HO-EPO-mEs having the double bond between the epoxy and hydroxy groups, such as 9-HO-12,13-*E*-EPO-10*E*-octadecenoate and/or 13-HO-9,10-*E*-EPO-11*E*-octadecenoate (Figure 6a_1_). Among their ^1^H NMR spectral signals, there is one near 3.09 ppm, due to one of epoxydic protons, and another one near to 5.94 ppm, due to the olefinic proton in *alpha* position in relation to the carbon atom that supports the OH group (Figure 6a_1_ and Table S3). These signals are in agreement with those reported by Gardner et al. [56] and Schieberle et al. [62]. In addition to the above-mentioned HO-EPO-mEs, other isomers such as 13-HO-9,10-*Z*-EPO-11*E*-octadecenoate (Table S3) could also be formed in this process because their signals at 3.07 (dt), 3.41 (dd) and 5.95 (dd) [63] are also present in the spectra of this oil from day 13 onwards. 

In the second group of HO-EPO-mEs, there are those having vicinal hydroxy and epoxy groups. Signals attributable to 11-HO-12,13-*E*-EPO-9*Z*-octadecenoate and to 11-HO-9,10-*E*-EPO-12*Z*-octadecenoate, such as those at 4.63 (dd) ppm of its methine carbinol proton, and at 2.98 (m) and 2.77 (d) ppm of the epoxydic protons [64] are observed in the spectra from day 13 onwards. Nevertheless, it should be mentioned that Ramsden et al. [25] assign to these compounds signals somewhat different from the above-mentioned (Table S3). Likewise, signals attributable to 11-HO-12,13-*E*-EPO-9*E*-octadecenoate and to 11-HO-9,10-*E*-EPO-12*E*-octadecenoate, such as those at 4.25 (dd) ppm of the methine carbinol protons in the erythro isomer or at 3.96 (q) ppm in the threo isomer, and signals of epoxydic protons at 2.93 (dtr) and 2.78 (dd) ppm [62,64,65,66], are also observable in the spectra (Table S3).

The concentration of the HO-EPO-mEs mentioned above belonging to the first group was estimated jointly using the area of the signal near 5.94–5.95 ppm. Regarding the second group of HO-EPO-mEs, due to the overlapping of signals, only the concentration of the threo isomers of 11-HO-12,13-*E*-EPO-9*E*-octadecenoate and of 11-HO-9,10-*E*-EPO-12*E*-octadecenoate was estimated using the area of the signal near 3.96 ppm. In both cases, it was assumed that these signals are due exclusively to the cited protons of this kind of compound. The evolution of the concentration of these compounds versus time is given in Figure 6b. It can be observed that concentrations of HO-EPO-mEs of the first group reach their maximum (near to 4.8 ± 1.2 mmol/mol TG) on day 14 and those of the second group of HO-EPO-mEs show increasing concentrations up to day 16 (near 3.7 ± 0.2 mmol/mol TG). 

It should be noticed that these compounds are also formed in living beings, and some of them have been associated with human psoriatic skin lesions [25]. Furthermore, some of these compounds have also been associated with the production of pain and itch in rodents’ skin [25]. In addition, it has been reported that 11-HO-12,13-*E*-EPO-9*Z*-linoleate and 11-HO-9,10-*E*-EPO-12*Z*-linoleate increase trigeminal neuron excitability, suggesting a potential role in headache or facial pain [67]. 

##### (f) Hydroxy-Keto-Monoenes (HO-KO-mEs)

**Figure 7:**
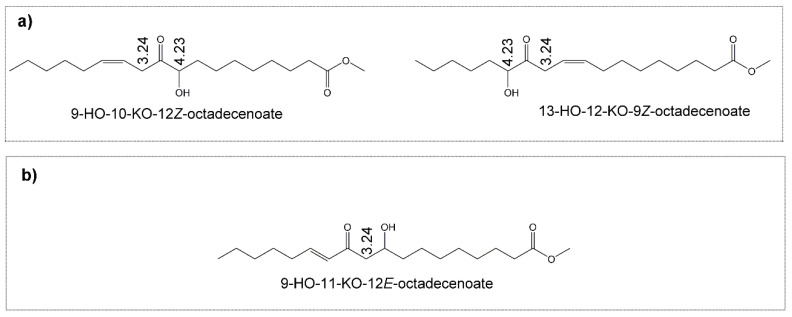
Chemical structures of the hydroxy-keto-monoenes (HO-KO-mEs) involved in this study, together with some chemical shifts (ppm) of the ^1^H NMR signals of some of their hydrogen atoms. (**a**) Structures in which keto group and the hydroxy group are on vicinal carbon atoms; (**b**) structure in which the hydroxy group is in *beta* position regarding the keto group.

These kinds of structures are also formed after 14 days under degradative conditions. Those found belong to two different groups. In one group, both oxygenated groups are on vicinal carbon atoms (Figure 7a), such as 13-HO-12-KO-9Z-octadecenoate and in 9-HO-10-KO-12*Z*-octadecenoate, with ^1^H NMR signals at 2.00 (m), 3.24 (t), 4.23 (dd), 5.54 (m) ppm [68], which are visible, with very low intensity, partially overlapped with other ones (assignments are indicated in Table S3). The other group of HO-KO-mEs is made up of structures that do not have the hydroxy and keto groups in vicinal carbon atoms. One example is 9-HO-11-KO-12*E*-octadecenoate, depicted in Figure 7b. Some of their ^1^H NMR signals, whose assignments are given in Table S3, appear at 2.58 (dd), 3.24 (d), 3.98-4.04 (m), 6.05 (dt) and 6.83 (dt) ppm [69]. These are also observable in the spectra of this oil after 14 days under oxidative conditions. The concentration of all these compounds is very small, between 1–2 mmol/mol TG, and it was estimated jointly, using the area of the signal at 3.24 ppm due to the methylenic protons in *alpha* position regarding either the keto group and the double bond, or regarding both oxygenated groups (Figure 7a,b), assuming that these signals are due exclusively to the cited protons of these kinds of compounds (Table S3). 

##### (g) Monoketo-Conjugated Dienes (mKO-c-dEs)

**Figure 8:**
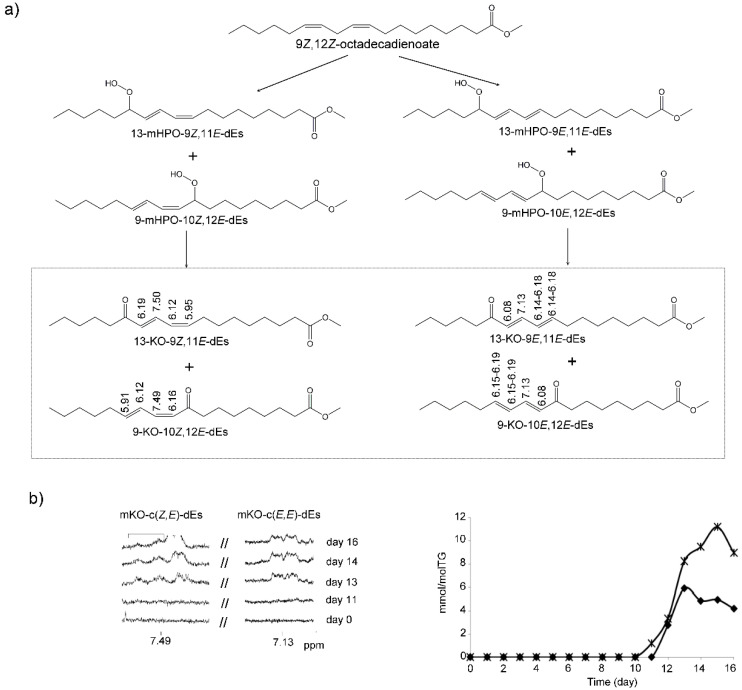
(**a**) Pathways of monoketo-conjugated dienes (mKO-c-dEs) formation proposed by some authors under certain oxidation conditions [61,70] together with some chemical shifts (ppm) of the ^1^H NMR signals of some of their hydrogen atoms. (**b**) Enlargements of some spectral regions where changes occur throughout the accelerated storage process and their evolution with time, together with the graphical representation of the evolution of the concentration, expressed as mmol/mol TG versus time given in days of mKO-c(*Z,E*)-dEs (♦); mKO-c(*E,E*)-dEs (*).

Monoketo-conjugated dienes, which are well-known oxylipins, were detected and quantified in this corn oil submitted to degradative conditions. Monoketo conjugated-*E,E*-dienes (mKO-c(*E,E*)-dEs) appear in the spectra from day 11 onwards and the *Z,E*-isomers (mKO-c(*Z,E*)-dEs) from day 12 onwards. Their formation has been described as coming from the corresponding monohydroperoxy-conjugated dienes (mHPO-c-dEs) [61] (Figure 8a). The identification of these compounds has been made taking into account ^1^H NMR spectral data given in the literature and with the use of standard compounds. The assignment of their signals is given in Figure 8a and in Table S4, and their quantification has been made using the area of signals centered near 7.49 ppm in the case of *Z,E*-isomers and centered near 7.13 ppm in the case of *E,E*-isomers. The concentration of the *E,E*-isomers is more than double than that of the *Z,E*-isomers (Figure 8b), and both are in line with that of their corresponding precursors mHPO-c(*E,E*)-dES and mHPO-c(*Z,E*)-dES at this time, but much lower. The maximum concentration of the *Z,E* isomers is reached on day 13 (near 5.0 ± 0.8 mmol/mol TG) and that of the *E,E* isomers on day 15 (near 11.2 ± 0.1 mmol/mol TG), decreasing slightly up to day 16 (Figure 8b). 

These results are of great interest because they show that monoketo-conjugated dienes are important secondary oxidation compounds derived from edible oils rich in linoleic groups, to which little attention has been paid until now, possibly due to the difficulty involved in their determination. Nevertheless, it should be noted that they have been attributed cytotoxicity, that of the *E,E*-isomers more than that of the *Z,E*-isomers [61,71].

##### (h) Keto-Epoxy-Monoenes (KO-EPO-mEs)

The formation of this kind of compound can be clearly observed in the spectra of this oil at advanced stages of the degradation process. Their signals appear from day 14 onwards, later than those of mKO-c-dEs, as may be expected. Bearing in mind the spectral signals, two groups of possible isomers are present.

**Figure 9:**
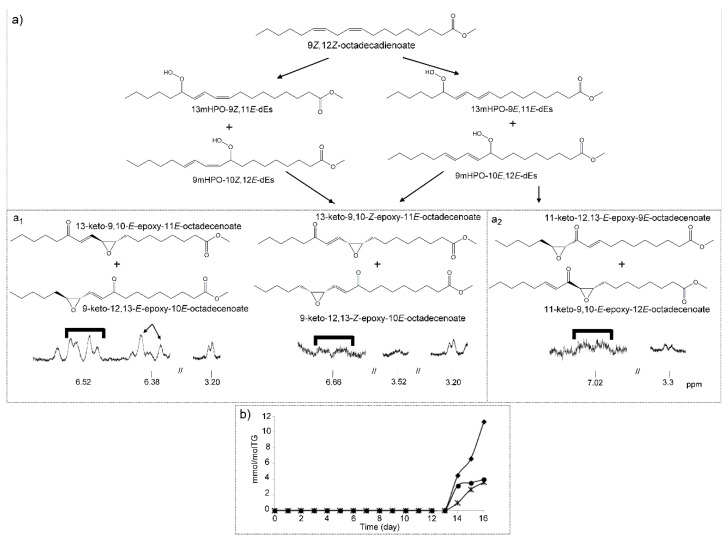
Pathways of formation of keto-epoxy-monoenes (KO-EPO-mEs) proposed by some authors under certain oxidation conditions [25,63,69]. (**a_1_**) KO-EPO-mEs having in their chains keto-double bond-epoxy, together with the enlargement of some regions of the ^1^H NMR spectra in which the signals of these compounds appear; (**a_2_**) KO-EPO-mEs having in their chains double bond-keto-epoxy together with the enlargement of some regions of the ^1^H NMR spectra in which the signals of these compounds appear. (**b**) Evolution of the concentration, expressed as mmol/mol TG versus time given in days of: 13-keto-9,10-*E*-epoxy-11*E*-octadecenoate + 9-keto- 12,13-*E*-epoxy-10*E*-octadecenoate (♦); 11-keto-12,13-*E*-epoxy-9*E*-octadecenoate + 11-keto- 9,10-*E*-epoxy-12*E*-octadecenoate (●); 13-keto-9,10-*Z*-epoxy-11*E*-octadecenoate + 9-keto-12,13-*Z*-epoxy-10*E*-octadecenoate (*).

One group of KO-EPO-mEs has the double bond between the keto and epoxy groups such as in 13-keto-9,10-*E*-epoxy-11*E*-octadecenoate and in 9-keto-12,13-*E*-epoxy-10*E*-octadecenoate. These structures have ^1^H NMR signals at 2.53 (t), 2.91 (td), 3.20 (dd), 6.38 (d) and 6.52 (dd) ppm. This group also includes 13-keto-9,10-*Z*-epoxy-11*E*-octadecenoate and/or 9-keto-12,13-*Z*-epoxy-10*E*-octadecenoate, which have ^1^H NMR signals at 2.55 (t), 3.20 (dd), 3.52 (dd), 6.40 (d) and 6.66 (dd) ppm. This has been confirmed with literature data [25,63,69] and with some standard compounds. Figure 9a_1_ gives the structures of these compounds, a potential formation pathway and the ^1^H NMR signals of some of their protons. The assignments of these signals are given in Table S4. The determination of their concentrations can be carried out using the intensity of several signals such as those that appear at 3.20 ppm, referring to both *E,E* plus *Z,E*-isomers; at 3.52 ppm, which refers to *Z,E*-isomers only; or at 6.38 ppm and 6.66 ppm due to *E,E*- and *Z,E*-isomers, respectively. The concentrations obtained and their evolution versus time are given in Figure 9b. Again, the concentration of *E,E*-isomers is higher than that of *Z,E*-isomers, in line with what was observed before in mHPO-c-dEs and mKO-c-dEs and also with the higher rate of decrease of the concentration of mKO-c(*E,E*)-dEs than of mKO-c(*Z,E*)-dEs at advanced oxidation stages (Figure 8b). These results demonstrate that these biologically potent oxylipins can be formed at advanced stages of the oxidation process of edible oils rich in omega-6 groups.

In addition, the formation in the oxidation of linoleic acid of other KO-EPO-mEs characterized because their oxygenated groups are supported on vicinal carbon atoms has also been described [25,69]. These compounds, like those above, come from mHPO-c-dEs and have been synthesized by either enzymatic or non-enzymatic pathways in vivo or in vitro from linoleic acid [69]. Signals attributable to this kind of compound, such as 11-keto-12,13-*E*-epoxy-9*E*-octadecenoate and/or 11-keto-9,10-*E*-epoxy-12*E*-octadecenoate, at 2.98–3.04 (ddd) ppm, 3.28–3.34 (d) ppm, 6.16–6.23 (dt) ppm and 7.02 (dt) ppm [69], are present, with very low intensity, in the ^1^H NMR spectra of this oil, after 14 days under degradative conditions (Figure 9a_2_). The assignment of these signals to the corresponding hydrogen atoms is indicated in Table S4. The appearance of these signals indicates that this kind of KO-EPO-mEs is also formed in this oil oxidation process. The estimation of their concentrations was made using the intensity of the signal at 7.02 ppm. Figure 9a_2_ shows their structure, and Figure 9b shows the evolution of their concentration in this process, which, as can be observed, is very low. 

The importance of the presence of these compounds in oxidized edible oils is great, due to their great reactivity. They are able to modify proteins through covalent bonds, have been related with numerous pathophysiological processes associated with inflammatory pain and respiratory diseases [72]. They have also been related with inflammation in skin [25] and with psoriatic lesions [25]. Moreover, it has been demonstrated that intradermal injection of these compounds induced itch-related scratching behavior in mice [25]. Finally, other authors have reported that some of these compounds stimulate aldosterone production [73], which is related to some types of human hypertension [74].

##### (i) Epoxy-Keto-Hydroxy Derivatives (EPO-KO-HOs)

**Figure 10:**
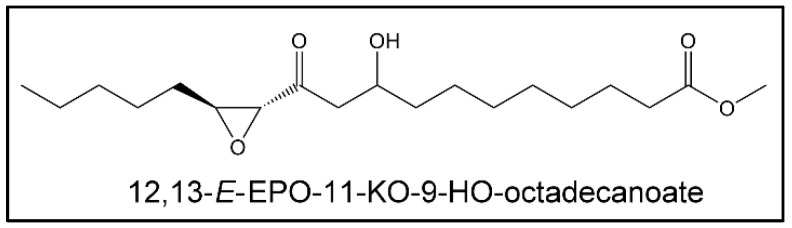
Chemical structure of epoxy-keto-hydroxy derivatives (EPO-KO-HOs), 12,13-EPO- *E*-11-KO-9-HO-octadecanoate.

From day 12 onwards under oxidative conditions, signals appear in the ^1^H NMR spectra of this oil near 2.42 (dd), 2.51 (dd), 3.02-3.08 (ddd), 3.16 (d) ppm and 3.98–4.04 ppm [69], which are overlapped with other ones. All these signals have been attributed to 12,13-*E*-EPO-11-KO-9-HO-octadecanoate (Figure 10) [69]. For this reason, it could be thought that structures with these three oxygenated functional groups could also have been formed in this oil. 

##### (j) Monoepoxy-Monoenes (mEPO-mEs) and Diepoxy (dEPO) Structures

The appearance in the oil spectra from day 13 of signals at 2.88–2.98 ppm and 2.66–2.73 ppm, together with other ones (Table S5), suggests the formation of monounsaturated epoxy structures (mEPO-mEs). Signals with these chemical shifts are due to protons of different kinds of monoepoxy derivatives. 

**Figure 11:**
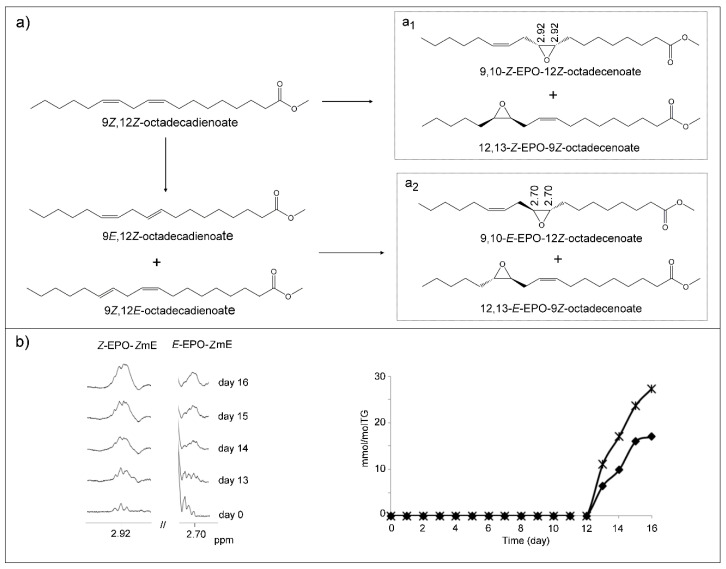
Pathway of formation of monoepoxy-monoenes (mEPO-mEs) proposed by some authors under certain oxidation conditions [75]. (**a_1_**) Formation of *Z*-EPO-mEs together with some chemical shifts (ppm) of the ^1^H NMR signals of their epoxydic hydrogen atoms; (**a_2_**) formation of *E*-EPO-mEs together with some chemical shifts (ppm) of the ^1^H NMR signals of their epoxydic hydrogen atoms. (**b**) Enlargement of regions of the ^1^H NMR spectra in which the signals of these compounds appear together with evolution of their concentration, expressed as mmol/mol TG versus time given in days of: *Z*-EPO-*Z*mEs (*); *E*-EPO-*Z*mEs (♦).

The first signal (2.88–2.98 ppm) and other ones present in the spectra agree with those of standard compounds such as 9,10-*Z*-EPO-12*Z*-octadecenoate (leukotoxin) and 12,13-*Z*-EPO-9*Z*-octadecenoate (isoleukotoxin) and also with those provided by Nilewski et al. [75] for the first compound. Likewise, there are other structures having epoxy groups whose protons give signals at this same chemical shift, which have been cited before. A pathway for the formation of this kind of epoxide [76] assumes that it is formed directly from intact linoleic chains with the participation of chains supporting hydroperoxy groups or peroxyl radicals (Figure 11a_1_). These latter could supply an oxygen atom to linoleic chains to form epoxy groups. At the same time, hydroperoxy groups or peroxyl radicals may evolve to give hydroxy groups or alkoxyl radicals, or keto groups [70]. In other words, these epoxides could derive directly from intact linoleic chains, although their formation would require the presence of hydroperoxides or peroxyl radicals, which would act as prooxidant systems. This reaction could lead to the formation of monoepoxides with the epoxy group just at the original position of the double bonds in the linoleic chain, forming leukotoxin or isoleukotoxin structures (Figure 11a_1_). These epoxidation reactions could be considered similar to those produced by oxidant systems such as hydrogen peroxide in acidic medium or by peroxycarboxylic acids [77,78,79]. All edible oils, including this one, contain acids as minor components [42], and the presence of high concentrations of hydroperoxides or of peroxyl radicals could give rise to this epoxidation reaction. It has also been proved that this kind of epoxide can be formed in cells. The concentration of *Z*-EPO-*Z*-mEs increases continuously from day 13, reaching its maximum value (27.3 ± 3.1 mmol/mol TG) on day 16 (Figure 11b). This was determined using the area of the signal at 2.88–2.98 ppm, subtracting the contribution of the above-mentioned structures that also give this signal. Both leukotoxin and isoleukotoxin can also be formed endogenously in cells and are known toxic compounds. They have been clinically associated with acute respiratory distress syndrome (ARDS), with circulatory shock and disseminated intravascular coagulation, and with multiple organ failure. This toxicity can be related to their great reactivity [80,81,82,83,84]. Nevertheless, it has been reported that these compounds, rather than being themselves toxic, could be considered as prototoxic, exhibiting toxicity in the presence of epoxide hydrolase, which opens the epoxy ring to give leukotoxin or isoleukotoxin diols, these latter being the toxic compounds able to provoke the above-mentioned diseases.

The second signal (at 2.66–2.73 ppm) mentioned above and other ones present in the spectra agree with those of the standard compound 9,10-*E*-EPO-12*Z*-octadecenoate and with those provided by Nilewski et al. [75] for 12,13-*E*-epoxy-9*Z*-octadecenoic acid. The pathway proposed for the formation of *E*-EPO-*Z*-mEs, as Figure 11a_2_ shows, involves an isomerization before the epoxidation reaction. The presence of these *E* epoxy structures is detectable in the ^1^H NMR spectrum from day 13 onwards and reaches maximum concentration (17.0 ± 1.6 mmol/mol TG) on day 16 (Figure 11b). This was determined using the area of the signal at 2.66–2.73 ppm, assuming that only the protons of this kind of compounds contribute to this signal. It should be noted that *Z*-EPO-*Z*-mE isomers are formed in greater concentration than *E*-EPO-Z-mEs isomers (Figure 11b). This may be because in the first case, a previous isomerization is not required. 

Finally, it could be thought that the formation of diepoxy structures (dEPO) could also occur in the oxidation process undergone by this oil, as described previously by other authors [79]. However, the absence of spectral signals of the standard, 9,10-EPO-12,13-EPO-octadecanoic acid, such as those at 2.98 and 1.73 ppm in the oil spectra throughout the oxidation process, raises doubts about this possibility. In Supplementary Material (Figure S2) are shown the following spectral regions in which these signals appear, of corn oil after 14 days under oxidative conditions; of the same sample enriched with 9,10-EPO-12,13-EPO-octadecanoic acid; and of pure 9,10-EPO-12,13-EPO-octadecanoic acid. It is evident that if diepoxides are formed in the oxidation process of this oil, they are in such very low concentrations that they cannot be detected by ^1^H NMR.

##### (k) Dihydroxy (dHO) and/or Polyhydroxy (pHO) Structures

**Figure 12:**
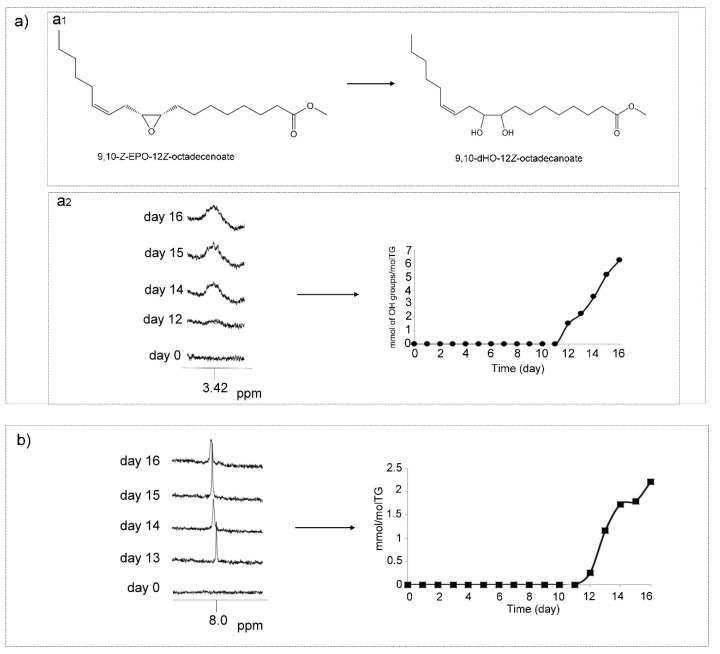
(**a**),(**a_1_**) Formation pathway of dihydroxy monoenes (dHO-mEs) proposed by some authors under enzymatic conditions [75]; (**a_2_**) Enlargement of the region of the ^1^H NMR spectra in which the signals of these compounds appear together with evolution of their concentration, expressed as mmol/mol TG versus time given in days; (**b**) Enlargement of the region of the ^1^H NMR spectra in which the signals of formic acid appear together with evolution of their concentration, expressed as mmol/mol TG *versus* time given in days.

The formation of dHO structures in the oxidation of this corn oil is also possible (Figure 12a_1_). In this study, as Figure 12a_2_ depicted, one broad signal centered at 3.42 ppm, attributable to the methine carbinol protons of 9,10-dHO-12*Z*-octadecanoate (leukotoxin diol) and/or 12,13-dHO-9*Z*-octadecanoate (isoleukotoxin diol) (Table S6) [75,85], appears in the spectra from day 12 onwards. For this reason, the presence of dHO structures cannot be discarded. Figure 12a_2_ represents the evolution of the concentration of methine carbinol protons that give this signal versus time after subtracting the contribution of HO-*Z*-EPO-*E*-mEs from day 13. The formation of hydroxy groups from epoxide ring opening, in acyl group chains of vegetable oils, can be produced by hydrolysis [86]. As already mentioned, dihydroxy structures, such as leukotoxin and isoleukotoxin diols, are known as toxic oxylipins, associated with multiple organ failure, and related to adult respiratory distress syndrome [80,87,88].

Furthermore, the formation of pHO can also occur in epoxidized triglycerides by oxirane ring opening by acids or by alcohols, as described previously [89], which will be commented on later. The methine carbinol proton of pHO could also contribute to the signal at 3.42 ppm. For this reason, data in Figure 12a_2_ also include these hydroxyl groups if there are any. 

##### (l) Acids and Formic Acid

It is known that all unoxidized edible oils contain fatty acids as minor components in small concentrations and also that, when they are submitted to oxidative conditions as here, a numerous group of acids of short chain length from formic to decanoic or further, are formed [10,32,90,91]. Of these, it has been reported that formic acid is formed in significant concentrations, and in fact several pathways have been proposed for its formation either as coming from recurrent oxidation of aldehydes [90,91] or even in the formation of 4-hydroperoxy-2*E*-nonenal from m-HPO-c-dEs [53]. How could it be otherwise in the oxidation process of this corn oil; the formation of formic acid has also been observed from day 12 onwards. This compound appeared simultaneously with some aldehydes, as will be commented on later. This was detected by the appearance in the ^1^H NMR spectra of the singlet signal at 8.01 ppm (Figure 12b and Table S6). The evolution of its singlet signal and of its concentration, estimated by ^1^H NMR throughout the oxidation process from day 12 up to day 16, are depicted in Figure 12b. The formation of this and other acids is of interest because they react with compounds which are present in the system to generate further derived compounds with important repercussions. 

##### (m) Poly-Formate (pF), Poly-Ester (pEst) and Poly-Hydroxy (pHO) Structures

**Figure 13:**
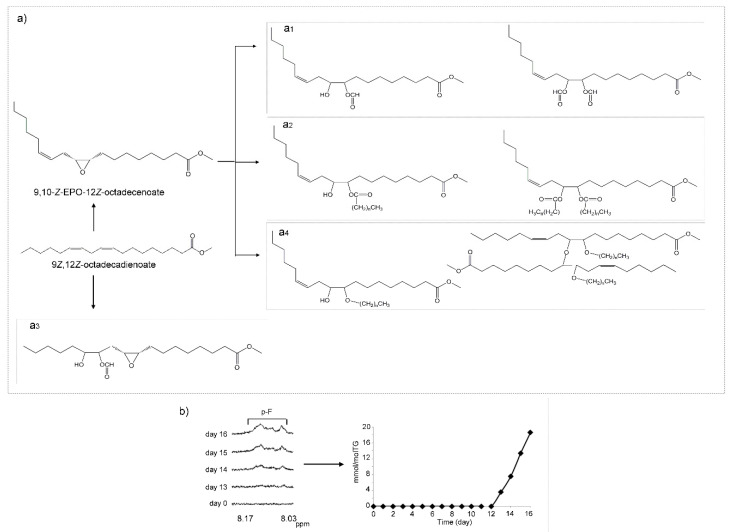
(**a**) Pathways of formation of poly-formates, poly-ester and poly-hydroxy structures (**a_1_**, **a_2_** and **a_3_**), and poly-ether and poly-hydroxy structures (**a_4_**), proposed by some authors [93,96,97]; (**b**) enlargement of region of the ^1^H NMR spectra in which the signals of poly-formates appear together with evolution of their concentration, expressed as mmol/mol TG versus time given in days.

In addition to the above-mentioned possibility of opening the oxirane ring to give diols, ring opening can also occur in the presence of acids. Formic acid is able to open the epoxydic rings, yielding one hydroxy group and one formate group as Figure 13a_1_ shows. The existence of this reaction in the oxidation process of this oil under the conditions of this study is confirmed by the appearance of signals between 8.03 and 8.17 ppm (Figure 13b and Table S6) of the proton of formate groups and at 5.18 ppm of methine protons of the ester group [92]. The appearance of formate signals in the spectrum occurs on day 13 and the concentration of these groups increases progressively up to the end of the polymerization process (Figure 13b). As mentioned above, other acids present in the oil could also open the oxirane rings in triglycerides forming poly-hydroxy and poly-ester groups (Figure 13a_2_) [93]. 

It should be added that it has also been described that epoxy, hydroxy and formate groups can be directly formed in the unsaturated chains of the oil triglycerides, in presence of formic acid and potent oxidants such as hydroperoxides, as Figure 13a_3_ shows. If the oxidative conditions are maintained, all epoxy groups can be opened to give more formate and hydroxyl groups [94]. In turn, some of the hydroxy groups formed can be esterified by formic acid (or by other acids) present, thus increasing the ratio between formate group (or ester group) and hydroxy groups. 

These reactions increase the viscosity of the oil, and to the best of our knowledge, they have not been previously described in the oxidation of edible oils, but rather in polymerization studies to obtain precursor of polymers based on edible oils. All oxidation compounds found in this oil and mentioned before can also be formed endogenously in cells, for which reason it could be thought that these poly-hydroxy and poly-formate structures could also be formed in cells and tissues, contributing to the loss of elasticity in membranes and even in blood vessels.

##### (n) Poly-Ether (pEt) and Poly-Hydroxy (pHO) Structures

It is known that not only acids are able to open the oxirane rings but so too can both primary and secondary alcohols [95]. This reaction can also take place during the oxidation of this oil because both kinds of alcohol groups are present. The occurrence of primary alcohols supported on small size molecules is well known in edible oils submitted to oxidation conditions [10,32]. Likewise, secondary alcohols supported on large-sized molecules such as the before-mentioned mono-hydroxy, di-hydroxy and even poly-hydroxy structures are also present. The oxirane ring opening provoked by alcohols yields, as Figure 13a_4_ depicts, one hydroxy group on the one side and on the other side one ether group, which incorporates into the molecule the structure that supports the alcohol group (Figure 13a_4_). Furthermore, if this reaction takes place between epoxides of one triglyceride and the secondary hydroxy groups of another triglyceride, polymerization is produced by the formation of C-O-C bridges between fatty chains. In addition, hydroxy groups of different structures may react with each other to generate ether groups. This reaction could also contribute to the polymerization of the sample.

From days 12–13 onwards, signals appear at near 3.62, 3.98 and 4.23 ppm, with increasing intensity to the end of the experiment, which can be assignable to methine protons of alcohols or to ether groups, in agreement with several authors. Thus, Caillol et al. [93] assign all signals comprised between 3.3 to 4.1 ppm either to methine protons of secondary hydroxyl groups or of ether bonds. De Souza et al. [98] also attribute the signal at 3.64 ppm to methine protons of secondary hydroxyl groups, and Lligadas et al. [97] to protons of the poly-ether backbone. Nevertheless, this signal could also be attributed to primary alcohols whose concentration increases, like acids, with oxidation time and are present in oxidized oils [32]. Likewise, the high increase observed in the intensity in the overlapped signals near 3.1 ppm, from day 12 onwards, could also be attributed to protons supported on carbon atoms of ether linkages.

As mentioned before concerning poly-esters, it could not be discarded that the poly-ether structures that produce polymerization in the oil could also be formed endogenously, contributing to the hardening of membranes, to the clogging of blood vessels and to the atherosclerotic plaque. 

##### (o) Structures Supporting Furan Ring (Frs)

**Figure 14:**
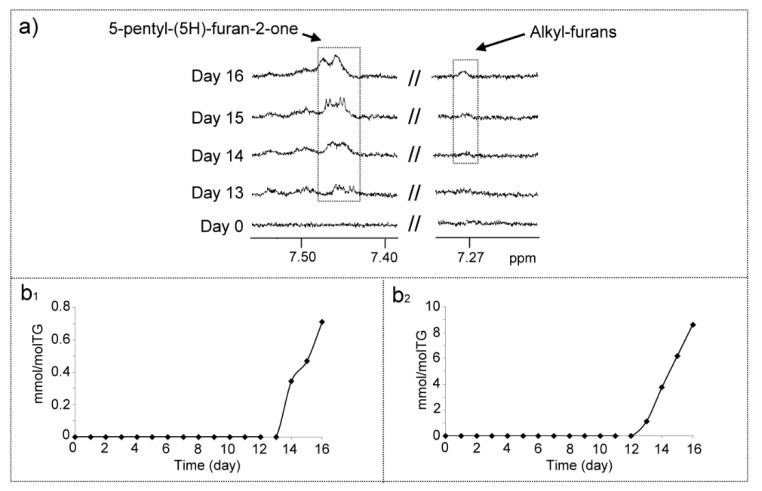
(**a**) Enlargement of a region of the ^1^H NMR spectra region in which the signals of alkyl-furans and 5-pentyl-(5H)-furan-2-one appear. (**b**) Evolution of the concentration of alkyl-furans (**b_1_**) and 5-pentyl-(5H)-furan-2-one (**b_2_**), expressed as mmol/mol TG versus time given in days.

Among these, two types can be distinguished, alkyl-furans and furanones. The formation of furan derivatives from methyl linoleate submitted to oxidative conditions has been described before [63]. Likewise, the formation of pentyl- and other alkyl-furans has also been reported previously in the oxidation of vegetable oils rich in diunsaturated acyl groups in several studies [10,22,23,32]. In this study, signals belonging to protons of alkyl-furans at 7.27 (dd) ppms appear (Figure 14a and Table S6) from day 14, growing in concentration up to the end of the experiment, as Figure 14b_1_ shows.

Likewise, ^1^H NMR signals attributable to 5-pentyl-(5H)-furan-2-one (Figure 14a and Table S6) and to other compounds of this family appear from day 13 onwards, with increasing intensity up to the end of the process. Among these there is a characteristic double doublet signal of their unsaturated protons centered near 7.47 ppm [99,100,101] that overlaps partially with that of m-KO-c(*Z,E*)-dEs, which appears one day before. These types of compounds have been found previously in several vegetable oils rich in diunsaturated acyl groups submitted to oxidative conditions [10,22,23,32]. Their formation has been described as coming from the fragmentation of hydroperoxy- and hydroxy-endoperoxides derived from polyunsaturated fatty acids [101]. The increasing concentration of these structures up to day 16 suggests that they are oxidation end products (Figure 14b_2_).

##### (p) Aldehydes (A)

Like hydroperoxides, aldehydes are well-known oxidation compounds. Several classical monitoring methods have been developed for their estimation over the years, such as *p*-Anisidine, but they do not provide accurate information. However, techniques such as ^1^H NMR spectroscopy allow the identification of the different kinds of aldehydes formed in oil oxidation processes and also their quantification [1,2,3,102], and SPME-GC/MS also permits the monitoring of the individually volatile aldehydes [32].

**Figure 15:**
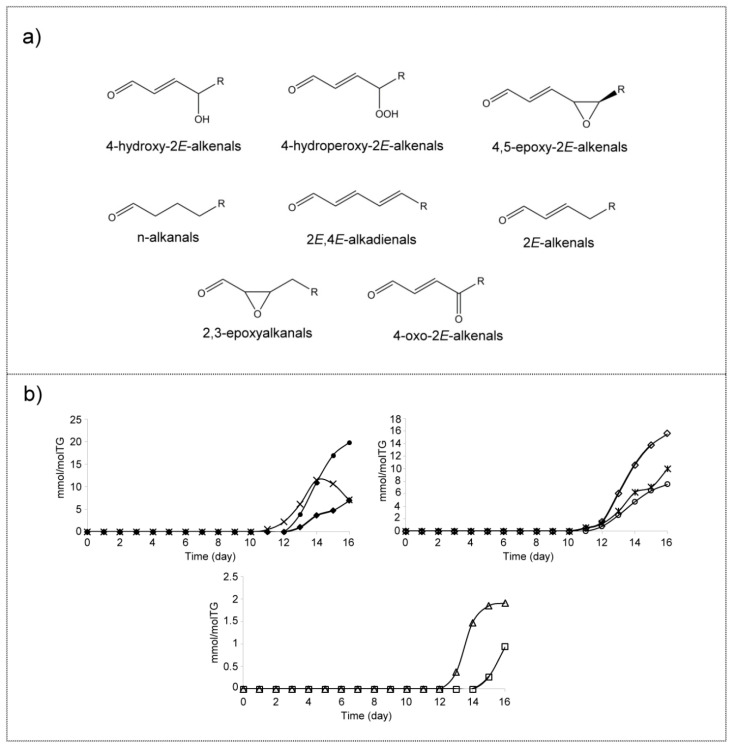
(**a**) Chemical structures of aldehydes detected in this study. (**b**) Evolution of their concentration, expressed as mmol/mol TG versus time given in days of: 4-hydroxy-2*E*-alkenals (●); 4-hydroxyperoxy-2*E*-alkenals (x); 4,5-epoxy-2*E*-alkenals (♦); 2*E*-alkenals (◊); 2*E*,4*E*-alkadienals (○); n-alkanals (*); 4-oxo-2*E*-alkenals (Δ); 2,3-epoxyalkanals (□).

The chemical shifts of the ^1^H NMR signals of the different kinds of aldehydes are given in Table S7. In the oxidation process undergone by this corn oil, the first aldehydes formed are 2*E*-alkenals and 4-hydroperoxy-2*E*-alkenals, which are detected from day 11 onwards. However, n-alkanals and 2*E*,4*E*-alkadienals appear in the spectra from day 12, whereas 4-hydroxy-2*E*-alkenals, 4-oxo-2*E*-alkenals and 4,5-epoxy-2*E*-alkenals appear on day 13. Finally, from day 15 the presence of 2,3-epoxyalkanals is observed (Figure 15a,b). It is of interest to note that the same functional groups (hydroperoxy, hydroxy, oxo (or keto) and epoxy) that are formed in the long chains of acyl groups are also present in these smaller aldehydic structures.

The structure of these aldehydes and the evolution of their concentration with time are given in Figure 15. It can be observed that the concentration of aldehydes reaches near 69 mmol/mol TG at the end of the experiment, the main ones being 4-hydroxy-2*E*-alkenals (19.8 ± 0.9 mmol/mol TG) and 2*E*-alkenals (15.6 ± 0.7 mmol/mol TG). All these detected structures can be supported either on small volatile molecules or on truncated acyl groups of TG and agree with findings of previous studies of edible oil oxidation [1,2,3,6,10,32,103].

The reactivity and toxicity of some of them such as those of oxygenated-*alpha,beta*-unsaturated as 4-hydroperoxy- and 4-hydroxy-2*E*-nonenal, as well as of 4,5-epoxy-2*E*-decenal among others, have been the subject of attention [20,22,33]. It must be remembered that these compounds have been held responsible for different degenerative diseases such as cancer, Alzheimer’s, or Parkinson’s among others [104,105,106].

Finally, it remains to be added that an important number of the above-mentioned compounds have been detected for the first time in oxidized edible oils in this study. Among them, there are important oxylipins such as dihydroperoxy-non-conjugated dienes (dHPO-nc-dEs), hydroxy-epoxy-monoenes (HO-EPO-mEs), hydroxy-keto-monoenes (HO-KO-mEs), some keto-epoxy-monoenes (KO-EPO-mEs) and *E*-epoxy-*Z*-monoenes (*E*-EPO-*Z*-mEs) as well as poly-formates, (or even other poly-esters), poly-ethers and poly-hydroxy structures. Some of these compounds had been detected and identified in previous studies as coming from oxidation of pure or standard compounds [50,54,56,62,63,68,69,75] or as in the case of poly-formates, poly-ethers and poly-hydroxy structures in the preparation of polymers derived from vegetable oils [92,96] but never in the oxidation of edible oils. There are other groups of compounds the presence of which in oxidized edible oils had been detected by other techniques but never by ^1^H NMR as in this study. In this group are formic acid, alkyl-furans, 5-pentyl-(5H)-furan-2-one, 4-oxo-2*E*-nonenal and 2,3-epoxyalkanals [10,22,23,32,90].

#### 3.2.2. Compounds Derived from Corn Oil Minor Components

As has been commented above, not only main but also minor components present in the oil undergo degradation under these conditions. Due to the low concentration of minor components, their derived oxidation compounds will be in very low concentration, a reason to expect that only a reduced number of them could be detected by ^1^H NMR spectroscopy.

**Figure 16:**
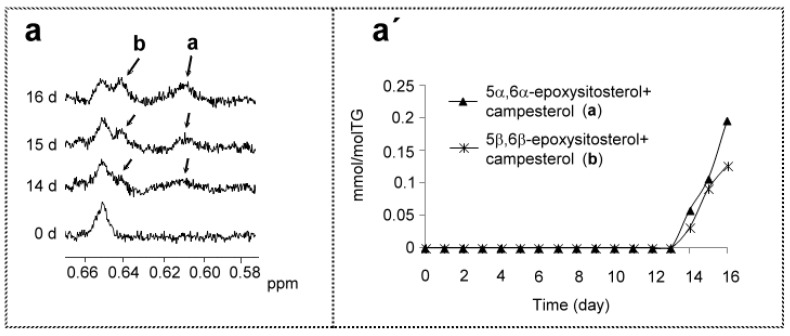
(**a**) Enlargement of region of the ^1^H NMR spectra region in which the signals of 5α,6α-epoxysitosterol+campesterol and 5β,6β-epoxysitosterol+campesterol appear. (**a′**) Evolution of their concentration, expressed as mmol/mol TG versus time given in days.

The main sterols of this oil are sitosterol+campesterol [42], and the only oxidation compounds derived from oil minor components detected are coming from them. They are 5α,6α-epoxysitosterol+campesterol and 5β,6β-epoxysitosterol+campesterol, which have singlet signals at 0.61 ppm and at 0.64 ppm respectively, in agreement with previous studies [107,108] (Table S8 and Figure 16a). They appear from day 14, in very low concentrations, 0.18 ± 0.04 mmol/mol TG and 0.14 ± 0.06 mmol/mol TG, for 5α,6α-epoxysitosterol+campesterol and 5β,6β-epoxysitosterol+campesterol, respectively, as Figure 16a′ shows. It should be pointed out that this is the first time that the presence of some sterols oxidation products has been detected directly in oxidized edible oil by the ^1^H NMR technique.

### 3.3. View of the Evolution over Time of the Oxidation Process

As mentioned, the process begins with the degradation of oil’s main and minor components. The evolution of the degradation of oil main components is well represented by that of linoleic groups. With this premise in mind, Figure 1b′ shows that four different stages can be observed in the process. In the first stage, the linoleic groups degradation is slow from day 0 to day 8 (rate of degradation is near −7.6 mmol/mol TG per day). In the second stage, from day 8 to day 11, the degradation is somewhat faster than in the first (near −28.9 mmol/mol TG per day are loss). In the third stage, from day 11 to day 14, the degradation reaches its highest rate (near −256.7 mmol/mol TG per day). Moreover, in the last stage that goes to day 16, the degradation rate remains high (−123.7 mmol/mol TG per day). At the end of the process, from the initial 1461.6 ± 0.1 mmol/mol TG of linoleic groups, near 291.2 ± 29.5 mmol/mol TG only remains without modification.

At the same time, the concentration of *gamma*-tocopherol, which is the main antioxidant of this oil, decreases at an almost constant rate from day 2 onwards (0.14 mmol/mol TG per day) up to day 11, being totally degraded on day 12 (Figure 1c′), just when the degradation of linoleic groups reaches the highest rate.

As linoleic groups are degraded, other structures are formed. The first to be formed are mHPO-c-dEs which can be detected and quantified from day 4 onwards (Figure 2b,c and Table S9). With detailed study of the concentrations, it is evident that the transformation of linoleic groups, up to day 8, is mainly due to the formation of mHPO-c-(*Z,E*)-dEs (rate of concentration increase of 3.3 mmol/mol TG per day) and of mHPO-c-(*E,E*)-dEs (rate of concentration increase of 3.9 mmol/mol TG per day). This stage coincides with the first stage of degradation of linoleic groups. In the following stage, from day 8 to day 11, the degradation rate of linoleic is somewhat higher than that of the increase of the concentration of mHPO-c-dEs, which is near 19.8 mmol/mol TG per day. From these, 4.7 mmol/mol TG per day are of mHPO-c-(*Z,E*)-dEs and near 15.1 mmol/mol TG per day are of mHPO-c-(*E,E*)-dEs). In other words, the increase in the concentration of mHPO-c-dEs (mmol/mol TG) represents only 68% of the mmol/mol TG of linoleic groups lost. This means that at least 32% of the linolenic groups degraded have generated other compounds that are not mHPO-c-dEs. This difference is much more marked in the following days, under degradative conditions, in such a way that from day 11 to day 13, the increase in the concentration of mHPO-c-dEs only represents 18% of the linolenic groups degraded in this period.

Likewise, it has also been proved that the increase in the concentration of hydroperoxy groups determined from the signal of the proton of the hydroperoxy groups between 8.3 and 9.3 ppm agrees exactly with that of mHPO-c-dEs from day 4 up to day 8. However, from this day onwards, the concentration of hydroperoxy groups is higher than that of mHPO-c-dEs. This fact proves that other hydroperoxy groups than these are present in the sample. Although the concentration of mHPO-c-dEs decreases strongly from day 13 onwards, as they are clearly intermediate compounds, they do not disappear totally from the sample, remaining to the end of the experiment in a significant concentration.

After mHPO-c-dE, as Table S9 shows, the following formed compounds detected by ^1^H NMR are mHO-c-(*Z,E*)-dEs. They appear on day 8, then reach very low concentration and disappear on day 13, showing their character of intermediate compounds. On day 9, dHPO-nc-*E,E*-dEs appear, presumably derived from mHPO-c-dE, and reach their maximum concentration on day 13, which diminishes, but they do not disappear, clearly showing their role as intermediate compounds. The formation of HPO-*E*-EPO-*E*-mEs is also fairly early; these compounds appear on day 10 and reach a maximum important concentration on day 14, decreasing slowly to day 16.

On day 11, in addition to m-KO-c(*E,E*)-dEs, two kinds of aldehydes appear in the sample, whose formation involves the break-up of the acyl group chain, namely 4-HPO-2*E*-alkenals and 2*E*-alkenals. These compounds reach their maximum concentration on days 15, 14 and 16 respectively, the concentration of the first two decreasing slightly at the end of the process (Table S9).

On day 12, there appear in the spectra, in addition to signals of m-KO-c(*Z,E*)-dEs, and of methine carbinol, protons of secondary alcohols which are either vicinal or not, signals of compounds formed from breaks in chains of acyl groups, such as n-alkanals, 2*E*,4*E*-alkadienals and formic acid. Except for the first structure (m-KO-c(Z,E)-dEs), which reaches its maximum concentration on day 13, the concentrations of the others increase up to the end of the experiment, albeit most of them in low concentrations, except the group due to the signal at 3.98 ppm, attributable to methine carbinol protons of secondary alcohols (Table S9).

A numerous group of different structures appears on day 13. Four of them reach important concentrations at the end of the experiment, as Table S9 shows. These are *Z*-EPO-*Z*-mEs, *E*-EPO-*Z*-mEs, pF and 4-HO-2*E*-alkenals. In addition to these, 5-pentyl-(5H)-furan-2-one, 4,5-EPO-2*E*-alkenals, 4-KO-2*E*-alkenals also appear on day 13 with increasing concentration to day 16. HO-*E*-EPO-*E*-mEs, HO-*Z*-EPO-*E*-mEs and *E*-EPO-KO-HOs also appear on day 13, reaching very low concentrations. Finally, on day 13, there appear signals at 3.62 and 4.24 ppm, associated to methine carbinol protons, in increasing concentrations up to the end of the process, probably associated with polymerized structures, although the first signal could also be attributed to primary alcohols, which are also formed in the oxidation of edible oils.

Day 14 also brings new oxidation compounds such as HO-*E*-EPO-*E*-mEs, HO-KO-*Z*-mEs, HO-KO-*E*-mEs, KO-*E*-EPO-*E*-mEs, KO-*Z*-EPO-*E*-mEs and alkyl-furans, all of them with increasing concentrations up to the end of the experiment, the concentration of KO-*E*-EPO-*E*-mEs being the main one. The last compounds to be detected were 2,3-EPO-alkanals on day 15 (Table S9).

## 4. Conclusions

^1^H NMR spectroscopy has allowed one, without chemical modification of the sample, to follow the oxidation process of this edible oil subjected to mild oxidative conditions in a global way over time. The estimation of the concentration of the main and minor components of the oil, its degradation kinetics and the identification and quantification of the new compounds formed during the oxidation process, as well as the estimation of the kinetics of their formation, and even of their further degradation, if this is the case, have been possible. As a consequence of the oxidation of the main components of this oil rich in polyunsaturated omega-6 groups, well-known oxylipins have been formed, such as hydroperoxy-, hydroxy- and keto-conjugated-dienes and different kinds of oxygenated-*alpha,beta*-unsaturated aldehydes such as 4-hydroperoxy-2*E*-nonenal, 4-hydroxy-2*E*-nonenal, 4-oxo-2*E*-nonenal and 4,5-epoxy-2*E*-decenal. It is noteworthy that these aldehydes are associated with different degenerative diseases such as cancer, Alzheimer’s or Parkinson’s, among others. In addition, and for the first time, the formation of an important number of other oxylipins, among which are dihydroperoxy-non-conjugated-dienes, hydroperoxy-epoxy-monoenes, hydroxy-epoxy-monoenes, keto-epoxy-monoenes, hydroxy-keto-monoenes, keto-hydroxy-epoxy-structures, and epoxy-monoenes has been proved. These findings are very important because most of these oxylipins have been found also in cells and have been related to several diseases, such as acute respiratory distress syndrome (ARDS), circulatory shock, disseminated intravascular coagulation and multiple organ failure. Furthermore, the formation of formic acid as well as of poly-formates (and probably of other poly-esters), poly-ethers and poly-hydroxy structures and of alkyl-furans and 5-pentyl-(5H)-furan-2-one has also been shown. The poly-formates, poly-esters, poly-ethers and poly-hydroxy structures are related to the increase of the oil viscosity and its polymerization degree. Although there are no previous reports, it could be thought that these latter oxidation compounds could also be formed endogenously and could be related to the hardening of cell membranes and the obstruction of blood vessels due to the increase in atherosclerotic plaque. Finally, it only remains to be added that the large volume of data obtained by means of ^1^H NMR spectroscopy have provided a global and depth insight on the oxidation process of this oil. All this information is extendable to the oxidation of other oils or systems rich in polyunsaturated omega-6 groups and is valuable for other researchers in future studies in which oxidation of this kind of lipids is involved both in food and in other fields of science.

## Supplementary Materials

The following are available online at https://www.mdpi.com/2076-3921/9/6/544/s1.
