# Supplementary material for: Oxylipins Associated to Current Diseases Detected for the First Time in the Oxidation of Corn Oil as a Model System of Oils Rich in Omega-6 Polyunsaturated Groups. A Global, Broad and in-Depth Study by 1H NMR Spectroscopy
Source: Antioxidants (Basel). 2020 Jun 20;9(6):544. doi: 10.3390/antiox9060544 (PMC7346112; doi:10.3390/antiox9060544)
Supplement: Supplementary file 1 [file antioxidants-09-00544-s001.pdf]

SUPPLEMENTARY MATERIAL

Oxylipins associated to current diseases detected for the first time in the oxidation of corn oil as a model system of oils rich in omega-6 polyunsaturated groups. A global, broad and in-depth study by ^{1}H NMR spectroscopy

J. Alberdi-Cedeño, María L. Ibargoitia, María D. Guillén*

Food Technology. Faculty of Pharmacy. Lascaray Research Center. University of the Basque Country (UPV-EHU). Paseo de la Universidad nº 7, 01006 Vitoria-Gasteiz, Spain, Telf: 34-945-013081, Fax: 34-945-013014.

E-mail: mariadolores.guillen@ehu.es*

Table S1. ^{1}H NMR signals, obtained in $CDCl_3$, of protons of main and of some minor corn oil components shown in Figures 1a, 1b, 1c and 1d, their chemical shifts, multiplicities and assignments to protons of different functional groups present in the corn oil. The signal letters agree with those given in the above mentioned Figures.

Signal	Chemical shift (ppm)	Multiplicity	Functional group	
		**Main components[a]**		
**A$_1$**	0.879	t	-C**H**$_3$	Saturated, monounsaturated ω-9 and/or ω-7 acyl groups
**A$_2$**	0.889	t	-C**H**$_3$	Unsaturated ω-6 acyl groups
**B**	0.972	t	-C**H**$_3$	Unsaturated ω-3 acyl groups
**C**	1.221-1.419	m	-(C**H**$_2$)$_n$-	acyl groups
**D**	1.522-1.700	m	-OCO-CH$_2$-C**H**$_2$-	acyl groups
**E1+E2**	1.941-2.139	m	-C**H**$_2$-CH=CH-	Monounsaturated ω-9 acyl groups<br>Unsaturated ω-6 acyl groups
**F**	2.305	dt	-OCO-C**H**$_2$-	Acyl groups
**G**	***2.765***	t	=HC-C**H**$_2$-CH=	Unsaturated ω-6 acyl groups
**TG**	***4.139, 4.303***	dddd	-C**H**$_2$OCOR	Glyceryl groups
**H**	5.225-5.296	m	>C**H**OCOR	Glyceryl groups
**I**	5.296-5.470	m	-C**H**=C**H**-	Acyl groups
		**Some minor components**		
**Δ7A[b]**	***0.540***	s	-C**H**$_3$ (C-18)	Δ7-avenasterol**
**STN[b]**	***0.651***	s	-C**H**$_3$ (C-18)	Sitostanol**
**S+C+Δ5A[b]**	0.684	s	-C**H**$_3$ (C-18)	β-sitosterol, campesterol and Δ5-avenasterol**
**ST+B[b]**	0.704	s	-C**H**$_3$ (C-18)	Δ5-stigmasterol and brassicasterol
**γ-T[c]**	***6.360***	s	-C**H** (Aromatic C-5)	γ-tocopherol**

Abbreviations: s: singlet; d: doublet; t: triplet; m: multiplet; dddd: double of double doublet.
*Area of the signals due to the protons in bold, were used for the quantification of each compound, using the equation [eq. 1] showed in the Materials and Methods.
**The assignment of the ^{1}H NMR signals of the protons was made with the aid of standard compounds.
***The assignment of the ^{1}H NMR signals of the protons was made with the data taken from the literature:
[a]Assignments of main components taken from:
Guillén, M.D., & Ruiz, A. (2003). *Eur. J. Lipid Sci. Tech. 105*(11), 688-696.
[b]Assignments of sterols-stanols taken from:
Sopelana, P., Arizabaleta, I., Ibargoitia, M.L., & Guillén, M.D. (2013). *Food Chem. 141*(4), 3357-3364.
Ibargoitia, M.L., Sopelana, P., & Guillén, M.D. (2014). *Food Chem. 165*, 119-128.
[c]Assignments of γ-tocopherol taken from:
Baker, J.K., & Myers, C.W. (1991). *Pharm. Res.* 8(6), 763-770.

Table S2. Chemical shift assignments and multiplicities of the ^{1}H NMR signals in $CDCl_3$ of protons of some hydroperoxides coming from the oxidation of main components detected in the corn oil during the oxidation process.

Chemical shift (ppm)	Multiplicity	Functional group	Compounds and/or family of compounds
Monohydroperoxides (mHPOs)			
8.48	dd	-OOH	
6.55[a]	dddd	-C**H**=CH-	9-hydroperoxy-10*E*,12*Z*-octadecadienoate
5.99	ddtd	-CH=CH-	13-hydroperoxy-9*Z*,11*E*-octadecadienoate
5.57	ddm	-CH=CH-	(mHPO-c(*Z,E*)-dEs)**
5.48	dtm	-CH=CH-	
8.42	br	-OOH	
6.24[a]	ddm	-C**H**=CH-	9-hydroperoxy-10*E*,12*E*-octadecadienoate
6.03	ddtd	-CH=CH-	13-hydroperoxy-9*E*,11*E*-octadecadienoate
5.72	dtm	-CH=CH-	(mHPO-c(*E,E*)-dEs)**
5.47	ddm	-CH=CH-	
Dihydroperoxides (dHPOs)			
5.36-5.50	m	-CH=CH-	9,12-dHPO-10*E*,13*E*-dE +
4.82[b]	dd	-C**H**-OOH	10,13-dHPO-8*E*,11*E*-dE
4.30-4.39	m	-CH-OOH	(dHPO-nc(*E,E*)-dEs)
2.33	t	-CH_2-	
Hydroperoxy-epoxy-monoenes (HPO-EPO-mEs)			
5.85[c]	dd	-C**H**=CH-	
5.47	dd	-CH=CH-	
4.33	m	-CH-OOH	9-HPO-12,13-*E*-EPO-10*E*-octadecenoate
3.11	dd	-HCOCH-	(HPO-*E*-EPO-mEs)
2.84	m	-HCOCH-	
2.30	t	-CH_2-	
Total hydroperoxides			
8.3-9.3[a]	br	-OO**H**	Total hydroperoxide groups<br>Total-OOH

Abbreviations: d: doublet; t: triplet; m: multiplet, br: broad singlet, dddd: double of double doublet

[*] area of the signals due to the protons in bold, together with the area of the sn-1 and sn-3 signals of TG shown, in Table S1 and in Figure 1, were used for the quantification of each compound, using the equation [eq. 1] showed in the Materials and Methods.

[**]The assignment of the ^{1}H NMR signals of the protons was made with the aid of standard compounds.

[***]The assignment of the ^{1}H NMR signals of the protons was made with the data taken from the literature:

[a]Assignments of monohydroperoxides (mHPOs) taken from:

Guillén, M.D., & Ruiz, A. (2005a). *Eur. J. Lipid Sci. Tech. 107*(1), 36-47.

Guillén, M.D., & Ruiz, A. (2005b). *J. Sci. Food Agri. 85*(14), 2413-2420.

[b]Assignments of dihydroperoxides (dHPOs) taken from:

Zhang, W. (2008). Synthesis and Fragmentation Reactions of Linoleic Acid-Derived Hydroperoxides (Doctoral dissertation, Case Western Reserve University).

Zhang, W., Sun, M., & Salomon, R.G. (2006). *J. Org. Chem. 71*(15), 5607-5615.

[c]Assignments of hydroperoxy-epoxy-monoenes (HPO-EPO-mEs) taken form:

Gardner, H.W., Weisleder, D., & Kleiman, R. (1978). *Lipids*, *13*(4), 246-252.

Table S3. Chemical shift assignments and multiplicities of the ^{1}H NMR signals in $CDCl_3$ of protons of some hydroxy derivatives coming from the oxidation of main components detected in the corn oil during the oxidation process.

Chemical shift (ppm)	Multiplicity	Functional group	Structures
Monohydroxy-conjugated dienes (mHO-c-dEs)			
6.48[a]	dd	-C**H**=CH-	
5.97	dd	-CH=CH-	(*Z,E*)-conjugated double bonds associated with
5.66	dd	-CH=CH-	hydroxides (OH)
5.45	dt	-CH=CH-	mHO-c(*Z,E*)-dEs**
4.15	m	-CH-OH	
Hydroxy-epoxy-monoenes (HO-EPO-mEs)			
5.94[b]	dd	-C**H**=CH-	
5.54	ddd	-CH=CH-	9-HO-12,13-*E*-EPO-10*E*-octadecenoate /
4.13	m	-CH-OH	13-HO-9,10-*E*-EPO-11*E*-octadecenoate**
3.09	dt,br	-CHOHC-	(HO-*E*-EPO-*E*-mEs)
2.81	dt	-CHOHC-	
5.95[c]	dd	-C**H**=CH-	9-HO-12,13-*Z*-EPO-10*E*-octadecenoate /
5.54	ddd	-CH=CH-	13-HO-9,10-*Z*-EPO-11*E*-octadecenoate
3.41	dd	-CHOHC-	(HO-*Z*-EPO-*E*-mEs)
3.07	dt	-CHOHC-	
5.65[d]	dt	-CH=CH-	
5.32	dd	-CH=CH-	11-HO-12,13-*E*-EPO-9*Z*-octadecenoate /
4.63	dd	-CH-OH	11-HO-9,10-*E*-EPO-12*Z*-octadecenoate**
2.98	m	-CHOHC-	(HO-*E*-EPO-*Z*-mEs)
2.77	d	-OH-HC-CHOHC-	
5.54[e]	m	-CH=CH-	Erythro-11-HO-12,13-*E*-EPO-9*E*-octadecenoate/
4.25	dd	-CH-OH	Erythro-11-HO-9,10-*E*-EPO-12*E*-octadecenoate
2.92	m	-CHOHC-	(Erythro- HO-*E*-EPO-*E*-mEs)
2.78	dd	-CHOHC-	
5.78	dtr	-CH=CH-	
5.53	ddtr	-CH=CH-	Threo-11-HO-12,13-*E*-EPO-9*E*-octadecenoate
3.96[e]	q	-C**H**-OH	Threo-11-HO-9,10-*E*-EPO-12*E*-ctadecenoate
2.93	dtr	-CHOHC-	(Threo- HO-*E*-EPO-*E*-mEs)
2.78	dd	-CHOHC-	
Hydroxy-keto-monoenes (HO-KO-mEs)			
6.83[f]	dt	-CH=CH-	
6.05	dt	-CH=CH-	9-HO-11-KO-12*E*-octadecenoate
3.98-4.04	m	-CH-OH	(HO-KO-*E*-mEs)
3.24	d	C=O-CH_2-	
2.58	dd	-CH_2-	
5.54	m	-CH=CH-	9-HO-10-KO-12*Z*-octadecenoate /
4.23	dd	-CH-OH	13-HO-12-KO-9*Z*-octadecenoate**
3.24[g]	t	C=O-C**H**$_2$-	(HO-KO-*Z*-mEs)
2.00	m	-CH_2-	

Abbreviations: s: singlet; d: doublet; t: triplet; m: multiplet, br: broad singlet, ddd: double of double doublet; q: quadruplet.

*area of the signals due to the protons in bold, together with the area of the sn-1 and sn-3 signals of TG shown, in Table S1 and in Figure 1, were used for the quantification of each compound, using the equation [eq. 1] showed in the Materials and Methods.

**The assignment of the ^{1}H NMR signals of the protons was made with the aid of standard compounds.

***The assignment of the ^{1}H NMR signals of the protons was made with the data taken from the literature:

[a]Assignments of mHO-c(*Z,E*)-dEs taken from:

Manini, P., Camera, E., Picardo, M., Napolitano, A., & d'Ischia, M. (2005). *Chem. Phys. Lipids*, *134*(2), 161-171.

[b]Assignments of 9-HO-12,13-*E*-EPO-10*E*-octadecenoate / 13-HO-9,10-*E*-EPO-11*E*-octadecenoate taken from:

Gardner, H.W., Weisleder, D., & Kleiman, R. (1978). *Lipids*, *13*(4), 246-252.

Gardner, H.W., & Kleiman, R. (1981). *BBA-Lipid Lipid Met. 665*(1), 113-125.

Schieberle, P., Trebert, Y., Firl, J., & Grosch, W. (1988). *Chem. Phys. Lipids*, *48*(3-4), 281-288.

Ramsden, C.E., Domenichiello, A.F., Yuan, Z.X., Sapio, M.R., Keyes, G.S., Mishra, S. K., ... & Davis, J.M. (2017). *Sci. Sign. 10*(493), eaal5241.

[c]Assignments of 9-HO-12,13-*Z*-EPO-10*E*-octadecenoate / 13-HO-9,10-*Z*-EPO-11*E*-octadecenoate taken from:

Hidalgo, F.J., Zamora, R., & Vioque, E. (1992). *Chem. Phys. Lipids*, *60*(3), 225-233.

[d]Assignments of 11-HO-12,13-*E*-EPO-9*Z*-octadecenoate / 11-HO-9,10-*E*-EPO-12*Z*-octadecenoate taken from:

Gardner, H.W., Kleiman, R., & Weisleder, D. (1974). *Lipids*, *9*(9), 696-706.

Ramsden, C.E.; Domenichiello, A.F.; Yuan, Z.X.; Sapio, M.R.; Keyes, G.S.; Mishra, S.K.; Gross, J.R.; Majchrzak-Hong, S.; Zamora, D.; Horowitz, M. S.; et al. (2017). *Sci. Sign. 10*(493), eaal5241.

[e]Assignments of Erythro-11-HO-12,13-*E*-EPO-9*E*-octadecenoate / Erythro-11-HO-9,10-*E*-EPO-12*E*-octadecenoate and Threo-11-HO-12,13-*E*-EPO-9*E*-octadecenoate / Threo-11-HO- 9,10-*E*-EPO-12*E*-ctadecenoate taken from:

Gardner, H.W., Kleiman, R., & Weisleder, D. (1974). *Lipids*, *9*(9), 696-706.

Gardner, H.W., & Kleiman, R. (1981). *BBA-Lipid Lipid Met. 665*(1), 113-125.

Gardner, H.W., & Crawford, C.G. (1981). *BBA-Lipid Lipid Met. 665*(1), 126-133.

Schieberle, P., Trebert, Y., Firl, J., & Grosch, W. (1988). *Chem. and Phys. Lipids*, *48*(3-4), 281-288.

[f]Assignments of 9-HO-11-KO-12*E*-octadecenoate taken from:

Lin, D., Zhang, J., & Sayre, L.M. (2007). *J. Org. Chem. 72*(25), 9471-9480.

[g]Assignments of 9-HO-10-KO-12*Z*-octadecenoate / 13-HO-12-KO-9*Z*-octadecenoate taken from: Gardner, H.W., Kleiman, R., Christianson, D.D., & Weisleder, D. (1975). *Lipids*, *10*(10), 602-608.

Table S4. Chemical shift assignments and multiplicities of the ^{1}H NMR signals in $CDCl_3$ of protons of some keto derivatives coming from the oxidation of main components detected in the corn oil during the oxidation process.

Chemical shift (ppm)	Multiplicity	Functional group	Structures
**Monoketo-conjugated dienes (mKO-c-dEs)**			
7.13[a]	dm	-C**H**=CH- (C-11)	*(E,E)*-conjugated double bonds associated with ketodiene of linoleic acyl groups mKO-c(*E,E*)-dEs[**]
6.15-6.19	m	-CH=CH-(C-12,13)	
6.07	d	-CH=CH- (C-10)	
2.54	t	-CH_2-CO	
7.49[a]	ddd	-CH=C**H**- (C-11)	*(Z,E)*-conjugated double bonds associated with ketodiene of linoleic acyl groups mKO-c(*Z,E*)-dEs[**]
6.16	d	-CH=CH- (C-10)	
6.12	m	-CH=CH- (C-12)	
5.91	dt	-CH=CH- (C-13)	
2.54	t	-CH_2-CO	
**Keto-epoxy-monoenes (KO-EPO-mEs)**			
6.52	dd	-CH=CH-	13-keto-9,10-*E*-epoxy-11*E*-octadecenoate / 9-keto-12,13-*E*-epoxy-10*E*-octadecenoate[**] (KO-*E*-EPO-*E*-mEs)
6.38[b]	d	-CH=C**H**-	
3.20	dd	-HCOCH-	
2.91	td	-HCOCH-	
2.53	t	-CH_2-	
**6.66**[b]	dd	-C**H**=CH-	13-keto-9,10-*Z*-epoxy-11*E*-octadecenoate / 9-keto-12,13-*Z*-epoxy-10*E*-octadecenoate (KO-*Z*-EPO-*E*-mEs)
6.40	d	-CH=CH-	
3.52	dd	-HCOCH-	
3.20	dd	-HCOCH-	
2.55	t	-CH_2-	
**7.02**[c]	dt	-C**H**=CH-	11-keto-12,13-*E*-epoxy-9*E*-octadecenoate / 11-keto-9,10-*E*-epoxy-12*E*-octadecenoate (KO-*E*-EPO-*E*-mEs)
6.23-6.16	dt	-CH=CH-	
3.34-3.28	d	-HCOCH-	
3.04-2.98	ddd	-HCOCH-	
2.25	t	-CH_2-	

Abbreviations: s: singlet; t: triplet; d: doublet; m: multiplet; ddd: double of double doublet

[*]area of the signals due to the protons in bold, together with the area of the sn-1 and sn-3 signals of TG shown, in Table S1 and in Figure 1, were used for the quantification of each compound, using the equation [eq. 1] showed in the Materials and Methods.

[**]The assignment of the ^{1}H NMR signals of the protons was made with the aid of standard compounds.

[***]The assignment of the ^{1}H NMR signals of the protons was made with the data taken from the literature:

[a]Assignments of mono-keto-conjugated dienes (m-KO-c-dEs) taken from:

Dufour, C., & Loonis, M. (2005). *Chem. Phys. Lipids. 138*(1), 60-68.

[b]Assignments of 13-keto-9,10-*E*-epoxy-11*E*-octadecenoate / 9-keto-12,13-*E*-epoxy-10*E*-octadecenoate and 13-keto-9,10-*Z*-epoxy-11*E*-octadecenoate / 9-keto-12,13-*Z*-epoxy-10*E*-octadecenoate taken from:

Hidalgo, F.J., Zamora, R., & Vioque, E. (1992). *Chem. Phys. Lipids. 60*(3), 225-233.

Lin, D., Zhang, J., & Sayre, L.M. (2007). *J. Org. Chem. 72*(25), 9471-9480.

Ramsden, C.E.; Domenichiello, A.F.; Yuan, Z.X.; Sapio, M.R.; Keyes, G.S.; Mishra, S.K.; Gross, J.R.; Majchrzak-Hong, S.; Zamora, D.; Horowitz, M. S.; et al. (2017). *Sci. Sign.*, *10*(493), eaal5241.

[c]Assignments of 11-keto-12,13-*E*-epoxy-9*E*-octadecenoate / 11-keto-9,10-*E*-epoxy-12*E*-octadecenoate from:

Lin, D., Zhang, J., & Sayre, L.M. (2007). *J. Org. Chem. 72*(25), 9471-9480.

Table S5. Chemical shift assignments and multiplicities of the ^{1}H NMR signals in $CDCl_3$ of protons of some epoxy derivatives coming from the oxidation of main components detected in the corn oil during the oxidation process.

Chemical shift (ppm)	Multiplicity	Functional group	Structures
**Monoepoxy-monoenes (mEPO-mEs)**			
5.56-5.47	m	-CH=CH-	9,10-*E*-EPO-12*Z*-octadecenoate / 12,13-*E*-EPO-9*Z*-octadecenoate (*E*)-EPO-*Z*-mE**
5.42-5.33	m	-CH=CH-	
2.73-2.66[a]	m	-C**H**O**H**C-	
2.30	t	$-CH_2-$	
5.46-5.55	m	-CH=CH-	9,10-*Z*-EPO-12*Z*-octadecenoate / 12,13-*Z*-EPO-9*Z*-octadecenoate *Z*-EPO-*Z*-mE**
5.43-5.34	m	-CH=CH-	
2.98-2.88[a]	m	-C**H**O**H**C-	

Abbreviations: t: triplet; m: multiplet.

*area of the signals due to the protons in bold, together with the area of the sn-1 and sn-3 signals of TG shown, in Table S1 and in Figure 1, were used for the quantification of each compound, using the equation [eq. 1] showed in the Materials and Methods.

**The assignment of the ^{1}H NMR signals of the protons was made with the aid of standard.

***The assignment of the ^{1}H NMR signals of the protons was made with the data taken from the literature:

[a]Assignments of mono-epoxy-monoenes (m-EPO-mEs) taken from:

Nilewski, C., Chapelain, C.L., Wolfrum, S., & Carreira, E.M. (2015). *Org. Lett. 17*(22), 5602-5605.

Table S6. Chemical shift assignments and multiplicities of the ^{1}H NMR signals in $CDCl_3$ of protons of other oxidation compounds coming from the oxidation of main components detected in the corn oil during the oxidation process.

Chemical shift (ppm)	Multiplicity	Functional group	Structures
Dihydroxy (dHO)/polyhydroxy (p-HO)			
5.61-5.52	m	-C<u>H</u>=CH-	9,10-dHO-12*Z*-octadecanoate /
5.46-5.36	m	-C<u>H</u>=C<u>H</u>-	12,13-dHO-9*Z*-octadecanoate
<u>3.48-3.37</u>[a]	m	-OHC**<u>H</u>**-C**<u>H</u>**OH-	dHO-mE[**]<br>p-HO
Formic acid			
<u>8.01</u>[b]	s	**<u>H</u>**-COOH	Formic acid
**Formates or poly-formates (p-F)**			
<u>8.17-8.03</u>[c]	m	$-H_2C$-O-C**<u>H</u>**=O	Polyformates<br>pF[**]
**Furane groups (Frs)**			
<u>7.45</u>[d]	dd	-CH=C**<u>H</u>-** (ar.C-4)	
6.11	dd	-C<u>H</u>=CH**-** (ar.C-3)	5-pentyl-(5H)-furan-2-one
5.04	m	-C<u>H</u>- (ar.C-5)	
<u>7.27</u>	dd	O-C**<u>H</u>**=CH**-** (ar.C-5)	
6.24	m	-CH=C<u>H</u>**-**(ar.C-4)	Alkyl-furans[**]
5.94	m	-C<u>H</u>=C**-** (ar.C-3)	

Abbreviations: s: singlet; d: doublet; m: multiplet.

[*]area of the signals due to the protons in bold, together with the area of the sn-1 and sn-3 signals of TG shown, in Table S1 and in Figure 1, were used for the quantification of each compound, using the equation [eq. 1] showed in the Materials and Methods.

[**]The assignment of the ^{1}H NMR signals of the protons was made with the aid of standard compounds.

[***]The assignment of the ^{1}H NMR signals of the protons was made with the data taken from the literature:

[a]Assignments of dihydroxy monoenes (dHO-mEs) taken from:

Nilewski, C., Chapelain, C.L., Wolfrum, S., & Carreira, E.M. (2015). *Org. Lett. 17*(22), 5602-5605.

[b]Assignments of formic acid taken from:

Babij, N. R., McCusker, E. O., Whiteker, G. T., Canturk, B., Choy, N., Creemer, L. C., ... & Li, F. (2016). *Org. Process Res. Dev. 20*(3), 661-667.

[c]Assignments of poly-formates taken from:

Abdullah, B.M., Zubairi, S. I., Huri, H.Z., Hairunisa, N., Yousif, E., & Basu, R.C. (2016). *PloS one*, *11*(3), e0151603.

Harry-O'kuru, R.E., Biresaw, G., Tisserat, B., & Evangelista, R. (2016). *J. Lipids*, *ID 3128604,* 12.

[d]Assignments of 5-pentyl-(5H)-furan-2-one taken from:

Bonete, P., & Najera, C. (1994). *J. Org. Chem. 59*(11), 3202-3209.

Braukmüller, S., & Brückner, R. (2006). *Eur. J. Org. Chem. 2006*(9), 2110-2118.

Table S7. Chemical shift assignments and multiplicities of the ^{1}H NMR signals in $CDCl_3$ of protons of aldehydes (A) coming from the oxidation of main components detected in the corn oil during the oxidation process.

Chemical shift (ppm)	Multiplicity	Functional group	Compounds and/or family of compounds
9.00[a]	d	-C**H**O	2,3-epoxyalkanals (2,3-EPO-alkanals)
3.20	m	-HCOCH-	
3.10	dd	-HCOCH-	
9.49[b]	d	-C**H**O	2*E*-alkenals**
6.86	tt	CHO-CH=CH-	
6.11	qt	-CH=CH-	
2.32	q	-CH_2-	
9.52[b]	d	-C**H**O	2*E*,4*E*-alkadienals**
7.09	m	CHO-CH=CH-	
6.33	m	-CH=CH-	
6.08	dd	CHO-CH=CH-	
2.22	m	-CH_2-	
9.55[b]	d	-C**H**O	4,5-epoxy-2*E*-alkenals** (4,5-EPO-2*E*-alkenals)
6.55	dd	CHO-CH=CH-	
6.39	dd	CHO-CH=CH-	
3.33	dd	-HCOCH-	
2.96	td	-HCOCH-	
9.57[b]	d	-C**H**O	4-hydroxy-2*E*-alkenals** (4-HO-2*E*-alkenals)
6.82	dd	CHO-CH=CH-	
6.31	dddd	CHO-CH=CH-	
4.42	m	-CH-OH	
9.58[b]	d	-C**H**O	4-hydroperoxy-2*E*-alkenals ** (4-HPO-2*E*-alkenals)
9.30	br,s	-OOH	
6.81	dd	CHO-CH=CH-	
6.29	m	CHO-CH=CH-	
4.63	dd	-CH-OOH	
9.75[b]	t	-C**H**O	n-alkanals**
2.40	dt	-CH_2-	
9.77[b]	d	-C**H**O	4-oxo-2*E*-alkenals (4-KO-2*E*-alkenals)
6.87	d	CHO-CH=CH-	
6.78	dd	CHO-CH=CH-	
2.69	t	-C=OC**H**-	

Abbreviations: d: doublet; t: triplet; m: multiplet; br: broad singlet; dd: double doublet;

*area of the signals due to the protons in bold, together with the area of the sn-1 and sn-3 signals of TG shown, in Table S1 and in Figure 1, were used for the quantification of each compound, using the equation [eq. 1] showed in the Materials and Methods.

**The assignment of the ^{1}H NMR signals of the protons was made with the aid of standard compounds.

***The assignment of the ^{1}H NMR signals of the protons was made with the data taken from the literature:

[a]Data taken from:

Daiboun, T., Elalaoui, M.A., Thaler-Dao, H., Chavis, C., & Maury, G. (1993). *Biocatalysis*, *7*(4), 227-236.

[b]Data taken from:

Guillén, M.D., & Ruiz, A. (2004). *Eur. J. Lipid Sci. Tech. 106*(10), 680-687.

Guillén, M.D., & Ruiz, A. (2005a). *Eur. J. Lipid Sci. Tech. 107*(1), 36-47.

Guillén, M.D., & Ruiz, A. (2005b). *J. Sci. Food Agric. 85*(14), 2413-2420.

Goicoechea, E., & Guillen, M.D. (2010). *J. Agric. Food Chem. 58*(10), 6234-6245.

Table S8. Chemical shift assignments and multiplicities of the ^{1}H NMR signals in $CDCl_3$ of protons of some sterols oxidation products coming from the oxidation of minor components detected in the corn oil during the oxidation process.

Chemical shift (ppm)	Multiplicity	Functional group	Compounds
**Sterols oxidation products**			
0.61	s	-C**H**$_3$ (C-18)	5α,6α-epoxysitosterol + campesterol
0.64	s	-C**H**$_3$ (C-18)	5β,6β-epoxysitosterol + campesterol

Abbreviations: s: singlet.

[*]area of the signals due to the protons in bold, together with the area of the sn-1 and sn-3 signals of TG shown, in Table S1 and in Figure 1, were used for the quantification of each compound, using the equation [eq. 1] showed in the Materials and Methods.

[**]The assignment of the ^{1}H NMR signals of the protons was made as in previous studies (Zhang, X., Geoffroy, P., Miesch, M., Julien-David, D., Raul, F., Aoudé-Werner, D., & Marchioni, E. (2005). *Steroids*, *70*(13), 886-895).

Table S9. Some oxidation compounds or structures detected in corn oil submitted to accelerated storage conditions, together with their detection time (day), the moment (day) in which they reach maximum concentration and the maximum concentration reached (mmol/molTG).

**Compounds and / or structures**	**Time (day) of maximum concentration**	**Maximum concentration reached (mmol/molTG)**
Detected from day 4		
mHPO-c(*Z,E*)-dEs	13	48.5 ± 2.4
mHPO-c(*E,E*)-dEs	13	140.9 ± 3.4
Detected from day 8		
mHO-c(*Z,*E)-dEs*	13	4.0 ± 0.8
Detected from day 9		
dHPO-nc(*E,E)*-dEs	13	19.2 ± 0.2
Detected from day 10		
HPO-*E*-EPO-*E*-m*E*s	14	38.7 ± 0.6
Detected from day 11		
m-KO-c(*E,E*)-dEs*	15	11.2 ± 0.1
4-HPO-2*E*-alkenals*	14	11.5 ± 1.5
2*E*-alkenals*	16	15.6 ± 0.7
Detected from day 12		
m-KO-c(*Z,E*)-dEs*	13	5.0 ± 0.8
dHO-*Z*-mEs* / p-OH	16	6.3 ± 0.3
Formic acid	16	2.2 ± 0.1
2*E*,4*E*-alkadienals*	16	7.6 ± 0.1
n-alkanals*	16	10.0 ± 1.1
Signal at 3.98	16	14.2 ± 0.0
Detected from day 13		
HO-*E*-EPO-*E*-mEs*<br>HO-*Z*-EPO-*E*-mEs	14	4.8 ± 1.2
Z-EPO-Z-mEs*	16	27.3 ± 3.1
E-EPO-Z-mEs*	16	17.0 ± 1.6
Poly-formates (pF)*	16	18.6 ± 2.1
5-pentyl-(5H)-furan-2-one	16	8.6 ± 0.5
4,5-EPO-2*E*-alkenals*	16	6.9 ± 0.5
4-HO-2*E*-alkenals*	16	19.8 ± 0.9
4-KO-2*E*-alkenals*	16	1.9 ± 0.1
Signal at 3.62	16	10.2 ± 0.5
Signal at 4.23	16	40.3 ± 0.2
Detected from day 14		
Threo-HO-*E*-EPO-*E*-mEs	16	3.0 ± 0.7
HO-KO-*Z*-mEs*<br>HO-KO-*E*-mEs	15	1.8 ± 0.6
KO-*E*-EPO-*E*-mEs*	16	15.3 ± 2.0
KO-*Z*-EPO-*E*-mEs	16	3.6 ± 0.6
Alkyl-furans*	16	0.7 ± 0.1
Detected from day 15		
2,3-EPO-alkanals	16	0.9 ± 0.0

* Compounds with an asterisk were acquired commercially and used as standards for identification purposes.

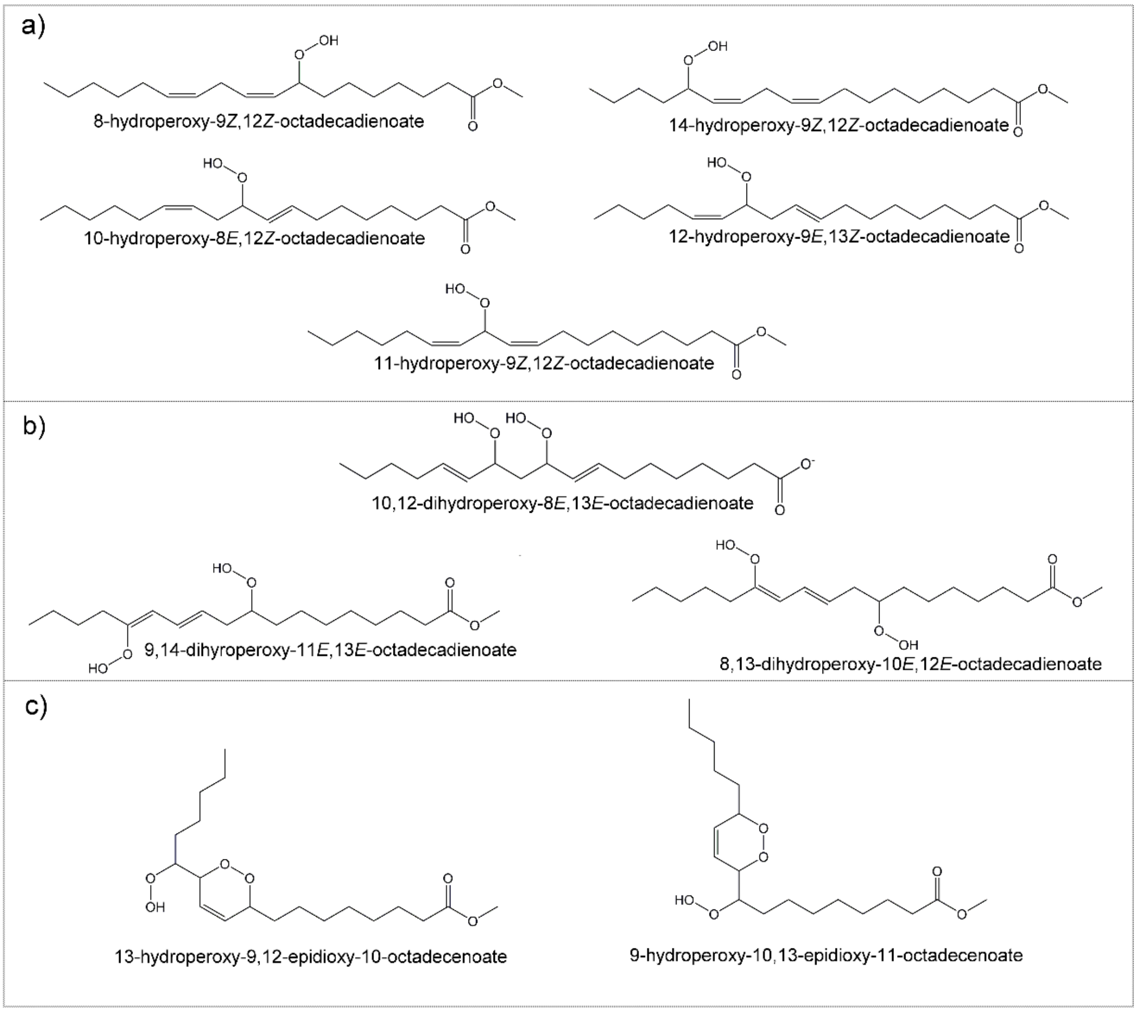

Figure S1. Chemical structures of some oxidation compounds having hydroperoxy groups in their structures, that could be formed during linoleic acyl groups oxidation but that have not been observed in the oxidation of corn oil under the conditions of this study, as commented on. **a)** monohydroperoxy-non conjugated dienes; **b)** dihydroperoxy-non conjugated dienes and dihydroperoxy-conjugated dienes; **c)** hydroperoxy-epidioxy-monoene.

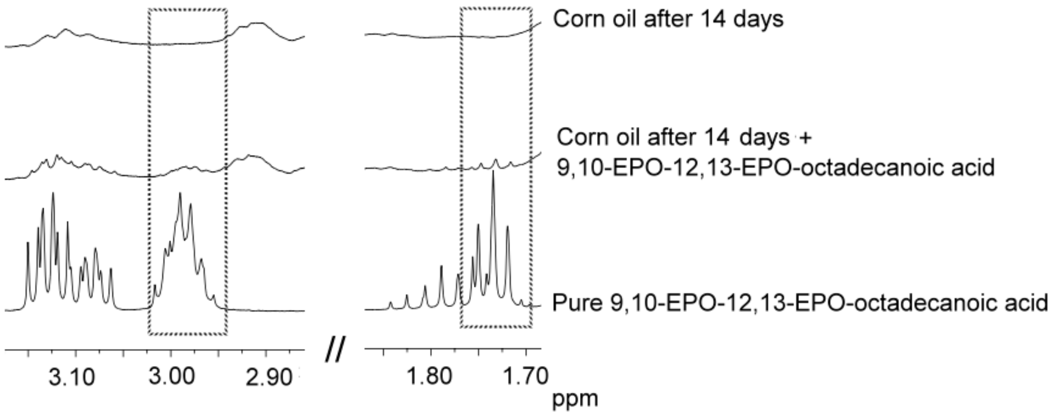

Figure S2. Enlargement of some regions of the ^1H NMR spectra of pure 9,10-EPO-12,13-EPO-octadecanoic acid, corn oil after 14 days under oxidative conditions enriched with 9,10-EPO-12,13-EPO-octadecanoic acid and corn oil after 14 days under oxidative conditions.
